# Evaluation of a new method for librarian‐mediated literature searches for systematic reviews

**DOI:** 10.1002/jrsm.1279

**Published:** 2017-11-28

**Authors:** Wichor M. Bramer, Melissa L. Rethlefsen, Frans Mast, Jos Kleijnen

**Affiliations:** ^1^ Medical Library—Erasmus MC Erasmus University Medical Centre Rotterdam Rotterdam the Netherlands; ^2^ Spencer S Eccles Health Sciences Library University of Utah Salt Lake City UT USA; ^3^ Department of Family Medicine, School for Public Health and Primary Care (CAPHRI) Maastricht University Maastricht the Netherlands; ^4^ Kleijnen Systematic Reviews Ltd York UK

**Keywords:** bibliographic databases, information storage and retrieval, quality improvement, review literature as topic

## Abstract

To evaluate and validate the time of completion and results of a new method of searching for systematic reviews, the exhaustive search method (ESM), using a pragmatic comparison.

**Methods:**

Single‐line search strategies were prepared in a text document. Term completeness was ensured with a novel optimization technique. Macros in MS Word converted the syntaxes between databases and interfaces almost automatically. We compared search characteristics, such as number of search terms and databases, and outcomes, such as number of included and retrieved references and precision, from ESM searches and other Dutch academic hospitals identified by searching PubMed for systematic reviews published between 2014 and 2016. We compared time to perform the ESM with a secondary comparator of recorded search times from published literature and contact with authors to acquire unpublished data.

**Results:**

We identified 73 published Erasmus MC systematic reviews and 258 published by other Dutch academic hospitals meeting our criteria. We pooled search time data from 204 other systematic reviews. The ESM searches differed by using 2 times more databases, retrieving 44% more references, including 20% more studies in the final systematic review, but the time needed for the search was 8% of that of the control group. Similarities between methods include precision and the number of search terms.

**Conclusions:**

The evaluated similarities and differences suggest that the ESM is a highly efficient way to locate more references meeting the specified selection criteria in systematic reviews than traditional search methods. Further prospective research is required.

## 1 INTRODUCTION

Systematic reviews are often challenged by retrieval bias, referring to failure in locating pertinent studies to include. To combat retrieval bias, comprehensive search methods are used to locate as many reports of studies as possible. Developing a comprehensive search for a systematic review can require large efforts.^1^, ^2^, ^3^ Estimates suggest that database searching may take an average of 17.7 hours.^4^ For each database used, it has been estimated that it may take an expert searcher 2 hours to translate a search adequately.^5^

There are well‐established suggestions for how to plan systematic review searches, particularly the recently produced Methodological standards for the conduct of new Cochrane Intervention Reviews and the standards of the National Academy of Medicine (formerly Institute of Medicine).^1^, ^6^ However, most guidelines do not specify how to construct a comprehensive search strategy within a database. In aggregate, librarian and information specialist‐authored search strategies are of higher quality. However, in practice, there is still variance between librarians and information specialists in comprehensiveness and overall search quality.^7^

Biomedical information specialists of Erasmus Medical Center of the Erasmus University in Rotterdam, the Netherlands (Erasmus MC), have created a blended approach to search strategy creation that combines objective methods of search term identification with librarian or information specialist expertise, here referred to as the “exhaustive search method,” or ESM.^8^ This method relies on creating single‐line search strategies in a text document and identifying relevant controlled vocabulary and free‐text terms by using database thesauri, an optimization technique to identify possibly relevant search terms, and macros to convert searches between different database syntaxes automatically.

The best method of comparing 2 or more search strategies is a prospective design, whereby alternative approaches are performed on a topic, the results pooled, and recall and precision calculated after the final included studies were chosen. Indeed, this study is underway. However, to provide initial validation of the ESM as a substitute for the traditional method, we sought a more pragmatic comparison with librarian‐mediated searches on a larger scale. The purpose of this study is to provide baseline evaluation and validation of the ESM search characteristics, precision, recall, retrieval, and speed.

## 2 METHODS

Since early 2013, librarian‐mediated searches by Erasmus MC information specialists were designed by using ESM. Embase is used as the primary search design interface, via http://embase.com. For each important element in the research question, thesaurus terms and synonyms for title or abstract searching are collected from the Emtree thesaurus. These terms are then combined into a single‐line search strategy in a Microsoft Word document by using predefined syntax formats.

To optimize sensitivity of the search, this preliminary search strategy is tested for completeness by using an optimization method. The method examines articles indexed with identified Emtree terms, but where the titles and abstracts lack the synonyms already used in the search strategy. Relevant terms from titles and abstracts are added to the search strategy, and their added value is evaluated in concert with the requesting researcher. Further optimization is done by reversing this process: looking for new thesaurus terms in articles where the titles and/or abstracts contain one of the identified synonyms but lack the already identified thesaurus terms.

Macros in MS Word translate the search strategies between platforms by replacing the syntax of one platform with the corresponding syntax of another platform. The standard procedure involves translation to MEDLINE via the Ovid interface, the Cochrane Library via Wiley Interscience, Web of Science Core Collection via ISI Web of Knowledge, and Google Scholar. Optional translation macros are available for Scopus and for CINAHL in the EBSCOhost interface. Using the macros, we make sure that there are no changes in the search terms for title and/or abstract. They remain constant between databases; only the thesaurus terms are changed between databases.

To understand whether searches performed by using the ESM differ from traditionally constructed searches, we sought to compare several attributes of complexity and time for librarian‐mediated searches conducted for systematic reviews. We compared systematic reviews published by Erasmus MC that were created by using the ESM, with systematic reviews published by other Dutch academic hospitals (DAHs) that were assisted by medical librarians. We selected DAH as a comparison for pragmatic reasons. First, biomedical information specialists in the Netherlands, in particular those in academic settings, regularly share their expertise in biannual meetings. The general level of search expertise among Dutch academic medical information specialists is therefore considered high and rather homogeneous. Furthermore, due to the small size of the group, most individuals are personally known to the first author, making it easier to recognize their involvement, even if they were mentioned only by name and not by function. Other studies examining librarian or information specialist involvement in systematic reviews have had difficulty identifying these contributors due to ambiguous names or reporting conventions.^7^, ^9^

To identify published systematic reviews for both the study and comparison groups, all systematic reviews published between 2014 and 2016 by DAHs, including Erasmus MC, were sought by using the search strategy below in PubMed. The search was last updated July 31, 2016.

(((Medisch Centrum[ad] OR Medical Hospital[ad] OR Medical Center[ad] OR Medical Centre[ad] OR umc[ad] OR mc[ad]) AND (leiden*[ad] OR maastricht[ad] OR utrecht[ad] OR groningen[ad] OR radboud[ad] OR nijmegen[ad] OR vu[ad] OR vrije universiteit[ad] OR free university[ad] OR amsterdam[ad])) OR AMC[ad] OR VUmc[ad] OR Radboudumc[ad] OR erasmus[ad] OR umcu[ad] OR umcg[ad] OR umcl[ad] OR lumc[ad]) AND (systematic review[tiab] OR systematically review*[tiab] OR systematic literature review*[tiab] OR systematic literature search*[tiab] OR systematic search*[tiab] OR systematically search*[tiab] OR medline[tiab] OR pubmed[tiab] OR embase[tiab] OR prisma[tiab] OR google scholar[tiab]) AND 2014:2016[dp].

After completing the search, we checked if each article that was retrieved reported the results of a systematic review. Protocols for the development of systematic reviews were excluded. We considered an article a systematic review if it met one or more of the following criteria:

- The title or abstract describes the study as a “systematic (literature) review.”
- The methods section describes that a systematic literature search was performed.
- The article was published in a source that primarily publishes systematic reviews (eg, Cochrane Database Syst Rev).
- The article is described in title or abstract as a review, and the process of article selection is presented in a PRISMA flow chart.

If the first or corresponding author's affiliation was Erasmus MC, we verified that the search strategy used for this review was librarian‐mediated by comparing Erasmus MC authors with our customer registration system. We excluded reviews where the original search was designed before January 1, 2013, as our method was developed in 2012. Articles meeting these criteria constitute the ESM group. From these identified systematic reviews, we collected the final number of references meeting their inclusion criteria and information on the topic of the review. Precision of the searches was calculated dividing the published number of final included references with the number of retrieved, deduplicated references from our search registration. Systematic reviews not reporting any relevant, retrieved references were excluded from precision calculations.

To be included in the comparison set, the first author had to be affiliated with a DAH other than Erasmus MC. Second, we included only articles where the assistance of a professional information specialist from the institution's medical library was mentioned in the full text. For the systematic reviews meeting these criteria, the number of databases and the names of the databases that were searched, the number of references found in MEDLINE, the total number of retrieved references after deduplication, the number of articles reviewed in full text, and the number of final included references were documented from the full text. We determined the number of search terms from the online appendices or full text. For that, we copied the complete MEDLINE Ovid or PubMed search strategy into an empty Microsoft Word document. For PubMed searches that had used field codes for each synonym, we simply documented the number of instances of an opening square bracket (“[”), which is a mark of the field codes in PubMed. For MEDLINE Ovid and for PubMed searches where field codes had not been used for all search terms, we used the Find function for each Boolean and proximity operator, summing the number of instances shown and adding the highest line number of the multiline search strategy to that total. We calculated the precision of the search as above by using published data. Because we did not restrict the comparison set to articles where all attributes were fully reported, we compared only those articles where data were available for each attribute individually.

Starting in November 2013, for each novel ESM study, we registered the actual search time of the Erasmus MC information specialist involved, starting at the beginning of the reference interview and ending when the searches for all databases were finalized. Our registrations did not include the time needed to import results in reference management software and to deduplicate them. We also did not count time needed for handsearching, reference checking, contacting key authors, or searching gray literature, as these tasks, in our institute, are generally performed by the review authors. When updates of the literature were needed, we only added extra time if changes had to be made to the search strategy; otherwise, the tasks were merely rerunning the searches and importing in reference management software, tasks that are not included in the time registration. Some ESM studies partially relied on reusing search elements from other searches or published filters; using time data from these studies may have inordinately lowered the average needed time for searches. These studies were not included in the ESM time study group, as we felt that in these cases, the time recorded would not be a good representation of the actual time needed to create such a complex search strategy.

Because data on the time needed to create the search strategies in DAH systematic reviews were unavailable, as a secondary comparison, we collated data from several published studies describing the time needed to create searches for systematic reviews.^4^, ^10^, ^11^, ^12^, ^13^, ^14^ We contacted the authors for detailed information about individual systematic review projects when it was not clear from the published papers. These individual data per review were then pooled in an MS Excel file, where we calculated quartiles and median values.

We analyzed the nature of the reviews' topics by using the tree structure of the MeSH databases. We searched in the MeSH database for the appropriate MeSH term for the disease described in the article and selected the corresponding high‐level MeSH term directly below the Diseases Category in the MeSH thesaurus. For the intervention element, we chose the appropriate top‐level MeSH term directly below the Analytical, Diagnostic, and Therapeutic Techniques and Equipment Category. If the most appropriate MeSH term for the intervention element fell within the top‐level MeSH term Therapeutics, we documented a MeSH term 1 level deeper. We also documented the domain of the review. We identified 7 domains. Reviews on the effectiveness of a treatment were assigned the domain therapy; those on policies fell in the group management. Research on incidence and prevalence were documented as epidemiology and reviews on causes of diseases as etiology. Other domains we documented were prognosis, diagnosis, and prevention.

Significance of differences between numerical observations (such as the number of search terms or the number of included references) in the study data and the comparison were calculated by using a 2 sided Mann‐Whitney test. Binary data (such as research topics and database use) were compared by using a Chi‐square test in SPSS.^15^ Differences with *P* values smaller than 0.05 were considered significant.

We further validated our comparison data (DAH) by briefly comparing the outcomes of the DAH and ESM data with another dataset, which was obtained from research by Borah et al.^16^ They gathered data from finished and published reviews registered in PROSPERO. We compared their data, using only the 38 reviews where we observed the acknowledgement of a medical librarian or information specialist, or when 1 of the coauthors' affiliations is a library, to the DAH and ESM data using range and medians.

## 3 RESULTS

**Figure 1:**
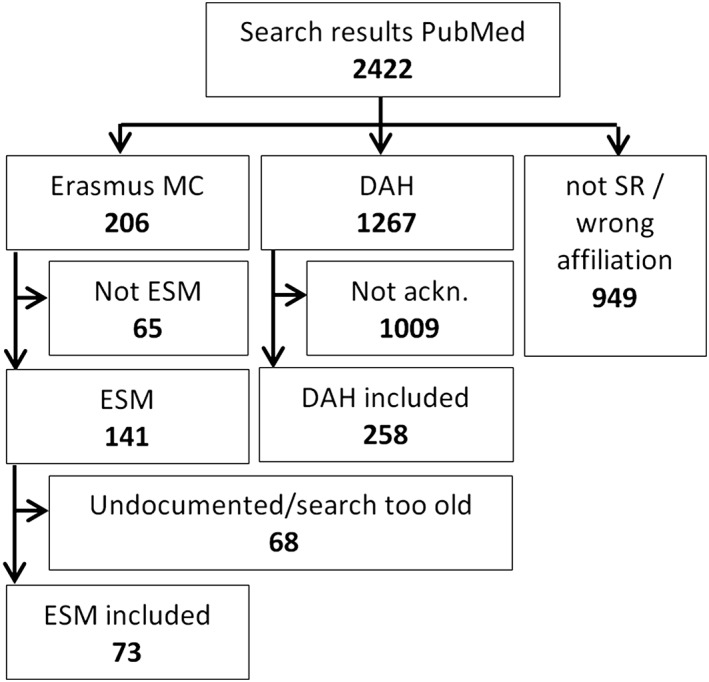
Flowchart of the inclusion exclusion process

On July 31, 2016, 2422 articles were reviewed. In 206 cases, the articles were systematic reviews with an Erasmus MC employee as first author. In 141 of these reviews, we identified that the search strategy was created by an Erasmus MC medical librarian. In 92 (65%) of these reviews, the assistance of a librarian was reported in the article. In 55 articles, the first author of this article (WMB) was acknowledged, 22 were coauthored by WMB, and in 20 articles, he was not mentioned by name, only by function. Sixty‐eight articles of the 141 either could not be traced in our registration, or the initial search was created before January 1, 2013. This left 73 articles in the ESM group for which all data were available. See supporting information file 1 for a complete overview of all included ESM publications and registered attributes. We found a total of 1271 systematic reviews where the affiliation of the first author was a DAH other than Erasmus MC. In 258 reviews (21%), the assistance of a medical librarian was reported. See supporting information file 2 for the references to the included DAH reviews. See Figure 1 for a flowchart of the inclusion/exclusion process.

Most reviews in the DAH group were written by researchers from Academic Medical Center (AMC) Amsterdam (93). Others came from Leiden University Medical Centre (53), VU Medical Centre Amsterdam (46), and Utrecht University Medical Centre (35). The DAH librarians acknowledged or coauthoring most frequently in the DAH reviews were a librarian from Leiden (20 acknowledgements/13 coauthorships), an information specialist from Utrecht (23 acknowledgements), and an AMC information specialist (7 acknowledgements/11 coauthorships). Of the 258 included reviews in the DAH group, 178 (68%) reported all attributes we collected. The most completely reported attribute was the number of databases searched (*n* = 256), while the lowest was the number of search terms used (*n* = 189). For each of the 73 reviews included in the ESM group, we documented all attributes, except the topics of the research question.

### 3.1 Topics of the research questions

There were some significant differences regarding the research questions in the 2 groups (see Table 1). No significant differences were observed in the patient/population or intervention elements of the reviews. The domain prognosis was overrepresented in the DAH group (*P* = .007) and epidemiology (*P* = .001) and etiology (*P* = .001) in the ESM group.

**Table 1:** Analysis of research questions between comparison (DAH) and study (ESM) group (only items where frequencies >5% are shown)

	DAH Comparison	ESM Group
Patient/Population	(N = 215)	(N = 63)
Neoplasms	19%	13%
Cardiovascular diseases	13%	14%
Wounds and injuries	7%	11%
Urogenital diseases	6%	11%
Mental disorders	7%	6%
Nutritional diseases	4%	10%
Musculoskeletal diseases	9%	3%
Otorhinolaryngology	7%	3%
Signs and symptoms	0%	6%
Skin diseases	5%	3%
**Intervention**	**(N = 128)**	**(N = 49)**
Surgical procedures	34%	31%
Chemicals and drugs	29%	35%
Diagnostic imaging	13%	6%
Physical therapy	0%	6%
**Domain**	**(N = 241)**	**(N = 68)**
Therapy	41%	35%
Prognosis*	24%	10%
Diagnosis	15%	9%
Management	12%	15%
Epidemiology*	4%	15%
Etiology*	2%	12%

* Significant difference (*P* < .05).

### 3.2 Databases searched

**Figure 2:**
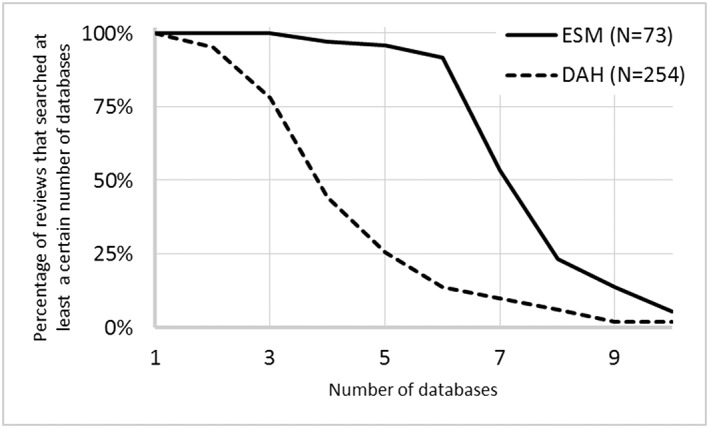
Number of databases searched

The number of databases searched was reported in 256 DAH systematic reviews (see Figure 2). Exhaustive search method systematic reviews searched more databases comparatively (ESM median = 7; DAH median = 3). There was a large variation between individual reviews (range: ESM = 3‐13; DAH = 1‐20). The difference is highly significant in favor of ESM (Mann‐Whitney *U* = 1959.5, *P* < .001).

The databases that were used significantly more frequently in the ESM reviews were Cochrane CENTRAL, Web of Science, Scopus, and, in particular, Google Scholar, which was used in 3% of the DAH reviews, but in 76% of the ESM reviews (for each of these 4 databases *P* < .001). See Table 2 for an overview of frequencies of database use.

**Table 2:** Frequency of use of different databases

	DAH (N = 254)	ESM (N = 73)
MEDLINE/PubMed	100%	100%
Embase	93%	100%
Cochrane CENTRAL*	56%	96%
CINAHL	29%	23%
Web of Science*	22%	89%
PsycINFO	20%	11%
Scopus*	3%	30%
Google Scholar*	3%	76%

* Significant difference (*P* < .05).

### 3.3 Search complexity

**Figure 3:**
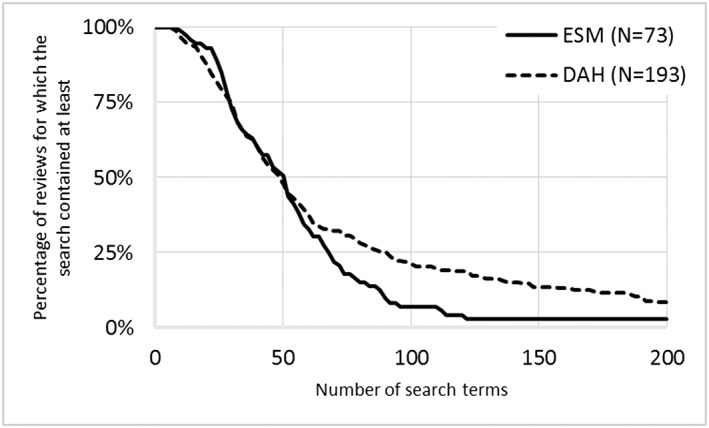
Number of search terms in the Medline search

For 193 (73%) DAH reviews, we could determine the number of search terms in the MEDLINE search strategy (see Figure 3). The DAH search strategies are more frequently very complex; for instance, 21% of DAH searches are more than 100 terms long, versus 7% in ESM searches. The medians were nearly equal, however (ESM = 50; DAH = 49). There is no significant difference (Mann‐Whitney *U* = 6517.5, *P* = .488).

### 3.4 Number of references retrieved

**Figure 4:**
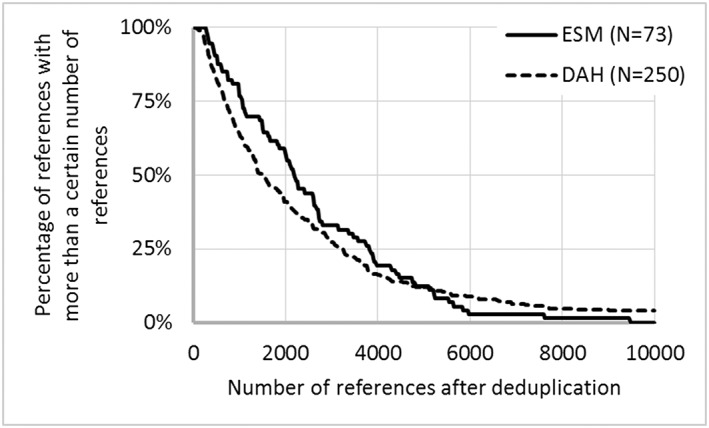
Total number of references retrieved after deduplication

The number of references after deduplication for all databases included in each review was reported in 250 (95%) DAH systematic reviews. Exhaustive search method searches retrieved more references after deduplication than DAH searches (see Figure 4). Although the average is similar (ESM = 2581; DAH = 2485), the median number of hits for ESM searches differed substantially (ESM = 2188; DAH = 1515). There was a great deal of variation between individual searches (range: ESM = 285‐9472; DAH = 74‐17,956). The difference is significant (Mann‐Whitney *U* = 7567.5, *P* = .041).

For 125 reviews in the DAH group, we could find the number retrieved for Medline. The median was lower for the ESM data (ESM = 875; DAH = 1074), but the difference was not significant (Mann‐Whitney *U* = 4150.5, *P* = .363).

### 3.5 Number of relevant references

In 243 reviews from the DAH group and in 62 of the ESM reviews, the number of articles screened in full text was mentioned. The median number of articles read in full text was higher in the ESM group (106) than in the DAH reviews (64). The difference is significant (Mann‐Whitney *U* = 8926.5, *P* = .024).

**Figure 5:**
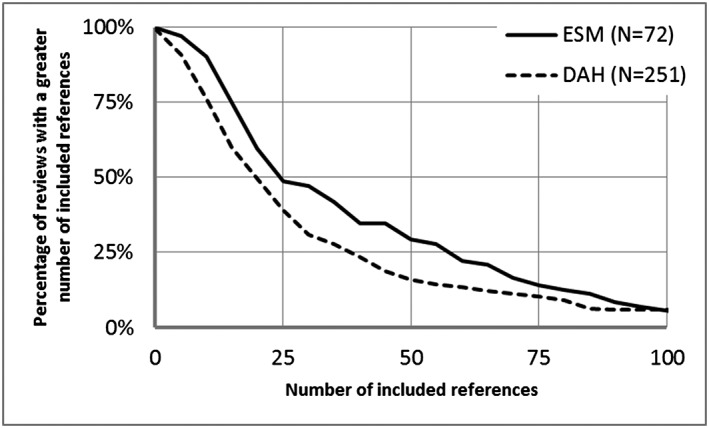
Number of included references

**Figure 6:**
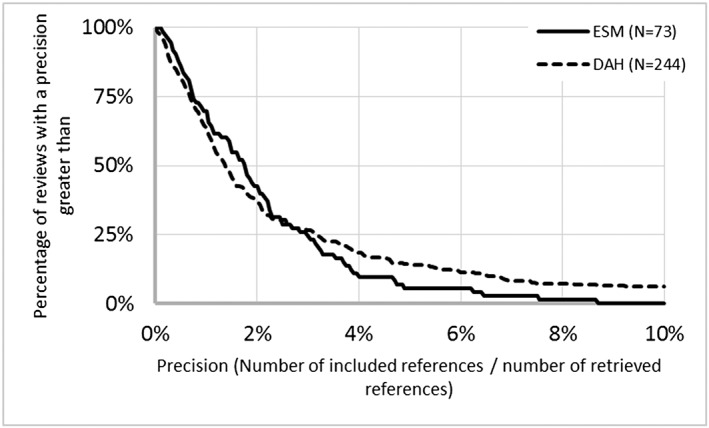
Precision (# included references/# retrieved references)

The final number of references meeting inclusion criteria was reported by 251 (98%) DAH systematic reviews (see Figure 5). Although there is a wide range (ESM: 4‐190; DAH: 0‐1342), the median was higher for ESM reviews (ESM = 25; DAH = 21) (see Figure 6). The difference is significant (Mann‐Whitney *U* = 10,951.5, *P* = .004).

### 3.6 Precision

Precision was calculable in 241 DAH systematic reviews (93%) where both the number of included references and the number of references after deduplication were reported, not including 1 review in the DAH group that reported they had retrieved no relevant references. The median precision for ESM was 1.8% compared with 1.4% for DAH (see Figure 6). There is no significant difference (Mann‐Whitney U = 8354.5, *P* = .515).

### 3.7 Time needed to create the search strategy

**Figure 7:**
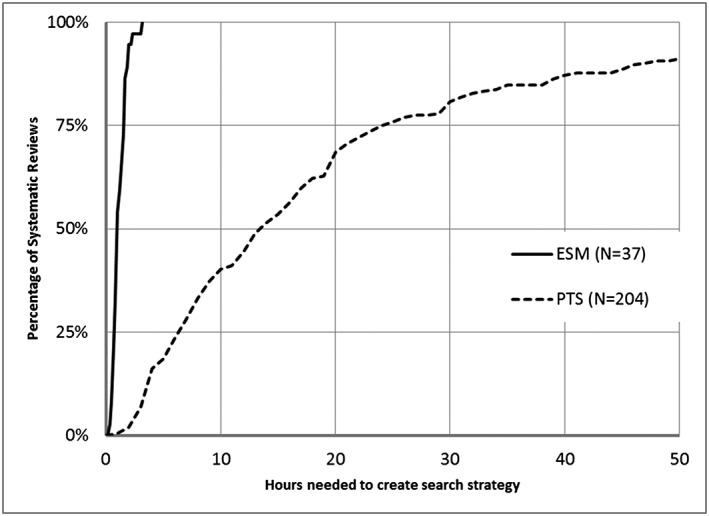
Time needed to create search strategies in hours

In 24 of the 72 included reviews in the ESM group, the initial searches were developed before November 2013, meaning before we began recording time needed to create the search strategies. In 11 cases where the search was developed after November 2013, we had reused parts of a search developed for another review; therefore, we had not been able to fully document the time needed to create the search strategy. For the remaining 37 (51%) ESM systematic reviews included in this research, the time needed to create the search strategies was registered at the time of search development. For the comparison data pooled from published time studies (PTS), we identified 105 published or unpublished systematic review projects.^4^, ^10^, ^11^, ^12^, ^13^, ^14^ These were combined with results from an online questionnaire in which information specialists were asked about the time they spent creating and translating searches for their last systematic review (N = 99).^17^ The results are compared in Figure 7. The median time needed in the pooled time studies was 12.8 hours, while the median time needed for ESM searches was 60 minutes. After 2 hours, the searches for 95% of all ESM reviews had been finalized compared with 2% of the PTS reviews. The difference is statistically significant in favor of ESM (Mann‐Whitney *U* = 466, *P* < .001).

## 4 DISCUSSION

Our baseline evaluation and validation of the ESM^8^ against pragmatic comparators of traditional librarian‐mediated searches reveal several differences and several similarities between outcomes. The ESM searches differed by using more databases, favoring the use of different databases (Web of Science, Scopus, Google Scholar), retrieving more references, including more studies in the final systematic review, and taking significantly less time to develop. Similarities between methods include similar precision and search complexity, as measured by number of search terms. These similarities and differences suggest that the ESM is a highly efficient way to locate more references meeting inclusion criteria than traditional search methods. Although ESM searches returned more results, which may require additional screening time, the searches' precision was equivalent to the DAH searches; meaning, the number needed to read to find studies for inclusion remains similar.

This initial evaluation validates our hypothesis that the outcomes of the ESM search method do not negatively compare to traditional search methods. In fact, using the ESM is a faster, more efficient way of searching more databases. The precision found by using both methods was statistically not different, although both groups demonstrated a lower median precision (ESM, 1.8%; DAH, 1.4%) than previously published research, which found a median precision of 2.9%.^18^ Because ESM did retrieve an overall higher median number of references, although the number of references in MEDLINE was lower than in ESM reviews than in the DAH group, it appears that ESM enables searchers to cast a wider net (searching more databases, eg) and find more relevant references that traditional searching methods are not able to locate.

Our validation study has several limitations. As noted above, we chose to utilize pragmatic, real‐world comparisons for our initial analysis rather than begin with a large‐scale prospective study. Our choice of using published systematic reviews from DAHs with librarian or information specialist‐mediated searches may be considered a weakness of our study design, as it is retrospective and not a random sample or a gold standard approach. However, several factors make this a reasonable choice and comparison. First, we included over 3 times as many articles in our DAH group as our ESM group, representing multiple hospitals and, more importantly, multiple searchers. Having multiple searchers, each of whom may have a slightly different approach to developing a search strategy, enables us to minimize the bias that may come from comparing single searcher to single searcher. Pragmatically, using DAHs also enabled us to easily determine whether a systematic review included a librarian or information specialist through name recognition.

To test the validity of our comparison data, we briefly compared the outcomes of the DAH and ESM data with another dataset, which was obtained from research by Borah et al.^16^ The number of included references in that set was lower than in both the DAH and ESM reviews (median 15, average 22). The number of databases searched lies between the DAH and ESM data (median: 5) as was the number of search terms (median: 52, average: 63). The number of articles retrieved was higher than both ESM and DAH data (median: 2615, average: 4981), and therefore, the precision was lower (median 0.5%, average 1.5%). Using our extra validation step, we ascertained that using the DAH group is reasonable; benchmark data from Borah et al did not show dramatic differences.

The DAH group's topical coverage did differ in certain aspects from coverage in the ESM group, which is a limitation of our study design. It was, however, impractical to compare all ESM reviews with matched topic reviews. Systematic reviews are generally executed on topics for which no other review exists. Matching topics in a broader sense would not increase the validity of the control group. We investigated whether differences in topics would account for the differences in number of included references. When comparing the number of included references within certain domains (such as therapy or epidemiology), we found that the median number of included references did not differ between domains.

Another factor potentially influencing the number of included references could be whether a review limited included references to study type. We found no significant difference in the percentage of reviews that limited to randomized controlled trials (6.6% for DAH, 5.6% for ESM). In the ESM reviews, we found that reviews that included only RCTs included fewer articles overall (15 compared with 29 in reviews that did not limit to RCTs); however, in the DAH reviews, the median number of included references when limited to RCTs was higher (23 compared with 20). We therefore reject the assumption that the topics of the reviews or study type limits were the main cause for the difference in number of included references.

A gold standard approach, as used to validate the Institute for Quality and Efficiency in Health Care (IQWiG) “objective approach,”^19^ can offer a stronger comparison, as search topics are compared head to head, but due to the complexity of creating gold standard sets from published systematic reviews, such studies are often limited in scope. The IQWiG analysis using this approach, for example, used a sample of 13 Cochrane reviews from which gold standards were created. For this study, they also assumed comprehensiveness of the search using a proxy of “a search in at least 2 bibliographic databases and 1 additional source (eg, a study registry) and documentation of the search strategy were required”.^19^ Because a gold standard set is inherently limited by the comprehensiveness of a search, and we do not consider a search in 3 resources to be necessarily comprehensive, our study instead attempted to look at broader trends, similarities, and differences between the results of our method and the traditional method.

A major difference between the DAH and ESM reviews is that the searches for all ESM reviews were performed by only 1 person, while the DAH reviews mentioned 29 searchers by name. Nine of these information specialists were involved in 10 or more of the DAH reviews, and 15 had contributed to 5 or more reviews. To account for the difference in experience, we checked whether the number of included references differed between reviews when searchers were involved in 5 or more reviews, compared with reviews where searchers were involved in 4 or fewer reviews in the DAH dataset. We did find a small difference in number of included references, but it was not significant (22 for experienced searchers; 19.5 for less experienced searchers, Mann‐Whitney *U* = 1892, *P* = .263). The median number of terms used by experienced information specialists was significantly higher than that of less experienced searchers (56 vs 41, Mann‐Whitney *U* = 807.5, *P* = .003).

A further limitation is our use of a second pragmatic comparator to assess time spent on creating the search strategies for systematic reviews. Because we believe that speed and efficiency are the major benefit of the ESM, it was a critical piece of our evaluation. Although we knew anecdotally that searchers spent far longer creating searches by using traditional methods than we did by using ESM, we were not able to ascertain the time spent creating the DAH searches retrospectively. Instead, we were required to establish a secondary comparator from published evidence and unpublished data from those studies, where necessary, to create a pool of 204 studies for which we had time data. Although this did not match our DAH data by design, the massive difference between time taken for ESM searches and the PTS searches is striking. The time difference would likely not be as striking for a novice using ESM, as indeed even ESM requires practice and expertise. Measuring time to complete a search is complicated by a myriad of factors. For instance, it is not always clear what is included as searching time in reported studies. Although we did not account for time spent searching for gray literature, deduplicating citations, handsearching, citation tracking, explaining the process of search strategy design to the researchers involved, or other tasks in our documentation for ESM searches, it is unclear whether some datasets of the comparison included these actions in their time logs. Saleh et al's study of time needed to search gray literature 4 did find that up to half of searching time could be spent searching gray literature. The data derived from a recent questionnaire among information specialists showed that, on average, 80% of the time for systematic reviews was spent searching bibliographic databases.^17^ We altered the PTS data for studies where it was not clearly defined what was measured in the recorded search time accordingly, or where this included more tasks than we registered: for those values, we used 80% of the reported time.

Although we found that ESM does work faster for the information specialist, because it finds more references, the extra retrieved references mean that the researchers have to spend more time screening. However, we believe that the extra time spent screening would improve the quality of the review, as more relevant references would be included. Researchers should not restrict the number of references to reduce the time to finish the review. However, information specialists have limited time and often large numbers of clients. Many institutions only employ 1 or 2 expert searchers. If the time needed per review for the searchers is reduced, this means they are able to provide more reviews with high quality search strategies. If the expert searcher would not have had time to create a search strategy for the review project, the review authors likely would have done the searches themselves, which would lead to the loss of relevant references. Therefore, the speed of ESM has potential to increase the overall quality of systematic reviews.

When comparing the number of final included references, we chose not to distinguish between references retrieved by database searches and by other methods (such as citation tracking, handsearching, and contacting key authors), in large part because most reviews do not specify where each reference was found. Some of the reviews in both groups have performed these extra activities; others have not. Sometimes, these differences were caused by an update of the search strategies by the authors. For example, in the review by Pieterman et al,^20^ 17 references were not found in our EndNote library, as they were of a later publication date than the last search date in our system. When correcting the data in the ESM results for the references not retrieved via our database searches, the number of included references showed only minor changes and the difference was still statistically significant. However, because we cannot similarly correct the DAH data, because we cannot easily distinguish where the DAH references were found, we kept all ESM final included references found outside database searches in our observations for parity.

We can only speculate why the ESM is so much faster than other methods but still able to provide good quality searches. Using single‐line search strategies allows for faster development and execution of search strategies. Searches can be easily adapted by adding extra terms without the need to regroup line numbers. Additionally, repeating a search strategy is much easier than having to meticulously type in all search statements in the correct order.^21^ The single‐line search strategies allow for optimization of the initial search strategy, which identifies extra relevant terms to be added to the search strategy that increase the sensitivity of the final search. The macros speed up the process of translating a search strategy across platforms and databases. Therefore, search strategies do not have to be built completely anew for each extra database.

The question remains whether ESM can be applied by other information specialists and other institutes. Our experience in teaching this method in workshops, even for experienced searchers, showed us that there might be a rather steep learning curve. One major problem that arises from single‐line search strategies is inconsistent use of parentheses. Mistakes are more easily made because it can be hard to see at a glance whether the number of opening and closing parentheses matches. This problem was nearly absent from our search development process because of our habit to immediately type a closing parenthesis when an opening parenthesis is typed, as well as the preparation of proximity operators, including all parentheses, before words are added. Additionally, other institutes are likely to have different subscriptions to databases and interfaces. We learned that the process is most efficient when using the interface of http://embase.com compared with using MEDLINE or Embase via Ovid. If other databases and interfaces are used, the macros we developed cannot be used in their current forms and they have to be adapted to the other institute's needs.

Although the ESM allows for quick search development, the method is not a guarantee for speed, as speed varies among users of the method. Speed is dependent on several factors, most notably experience with this method. The ESM benchmark times related in this study should not be used to set time limits on search strategy development. Instead, the method should be used to improve search strategy quality and over time can, as experience of the searcher with this method grows, result in time reduction per search.

This evaluation study is a first step toward validating the ESM. Further studies must be undertaken to provide stronger prospective evidence of the method's outcomes and speed compared with traditional searching methods. This research is currently underway. As with the IQWiG team's validation of their objective approach to searching by using a similar prospective design, 19 a prospective study will be necessarily smaller in scope than this more wide‐scale evaluation, although it will offer us a more robust study design.

## 5 CONCLUSIONS

The ESM currently used at Erasmus MC creates opportunities for faster development of systematic review search strategies that find more relevant studies than other methods with equivalent search precision. Using this method, librarians and information specialists can potentially help many more people with the development of exhaustive searches for systematic reviews than traditional search methods allow without loss of complexity and search precision.

Future studies should assess recall in addition to the attributes reported here. For a thorough comparison, one should perform the searches for 1 systematic review topic with multiple searchers, include all results for the reviewers to prepare their review, and then check whether each searcher retrieved all included references. Research is currently being undertaken on the ESM by using this prospective design.

## Author contributions

WB, FM, and JK developed the study concept. WB performed the searches and gathered and analyzed the data. MR checked the data. WB and MR drafted the manuscript. All authors critically revised the manuscript and gave final approval.

## Funding

Melissa Rethlefsen receives funding in part from the National Center for Advancing Translational Sciences of the National Institutes of Health under Award Number UL1TR001067. The content is solely the responsibility of the authors and does not necessarily represent the official views of the National Institutes of Health. The other authors declare no financial interest in this study.

## Supporting information

Appendix Benchmark 1: Included ESM systematic reviews

Appendix Benchmark 2: Included DAH systematic reviews

## References

[1] Institute of Medicine, Committee on Standards for Systematic Reviews of Comparative Effectiveness Research . Finding What Works in Health Care: Standards for Systematic Reviews. Washington, DC: The National Academies Press; 2011. 24983062

[2] Higgins JPT , Green S . Cochrane Handbook for Systematic Reviews of Interventions. West Sussex, UK: Wiley Online Library; 2008, DOI: 10.1002/9780470712184.

[3] Chandler J , Churchill R , Higgins J , Lasserson T , Tovey D . Methodological standards for the conduct of new Cochrane Intervention Reviews. 2013; Version 2.3:http://editorial-unit.cochrane.org/sites/editorial-unit.cochrane.org/files/uploads/MECIR_conduct_standards%202.3%2002122013_0.pdf. Accessed 21 December, 2015.

[4] Saleh AA , Ratajeski MA , Bertolet M . Grey literature searching for health sciences systematic reviews: a prospective study of time spent and resources utilized. Evid Based Libr Inf Pract. 2014;9(3):28‐50. 10.18438/B8DW3K 25914722 PMC4405801

[5] Erwin PJ . By the clock: how much time does an expert search take? MLA news. 2004;370:1,12

[6] MECIR. Methodological standards for the conduct of Cochrane intervention reviews. 2013; http://editorial-unit.cochrane.org/sites/editorial-unit.cochrane.org/files/uploads/MECIR_conduct_standards%202.3%2002122013.pdf. Accessed May, 7, 2015.

[7] Rethlefsen ML , Farrell AM , Osterhaus Trzasko LC , Brigham TJ . Librarian co‐authors correlated with higher quality reported search strategies in general internal medicine systematic reviews. J Clin Epidemiol. 2015;68(6):617‐626. 10.1016/j.jclinepi.2014.11.025 25766056

[8] Bramer WM , de Jonge GB , Rethlefsen ML , Mast F , Kleijnen J . An efficient approach to systematic literature searching. Accepted for publication in J Med Libr Assoc 2018. 10.5195/jmla.2018.283 PMC6148622 30271302

[9] Koffel JB . Use of recommended search strategies in systematic reviews and the impact of librarian involvement: a cross‐sectional survey of recent authors. PLoS One. 2015;10(5):e0125931):e0125931 10.1371/journal.pone.0125931 25938454 PMC4418838

[10] Allen IE , Olkin I . Estimating time to conduct a meta‐analysis from number of citations retrieved. JAMA. 1999;282(7):634‐635. 10.1001/jama.282.7.634 10517715

[11] Gann LB , Pratt GF . Using library search service metrics to demonstrate library value and manage workload. J Med Libr Assoc. 2013;101(3):227‐229. 10.3163/1536-5050.101.3.015 23930096 PMC3738086

[12] Hausner E , Guddat C , Hermanns T , Lampert U , Waffenschmidt S . Prospective comparison of search strategies for systematic reviews: an objective approach yielded higher sensitivity than a conceptual one. J Clin Epidemiol. 2016;77:118‐124. 10.1016/j.jclinepi.2016.05.002 27256930

[13] Nauche B , Landry T . Implanter un service de soutien aux revues systématiques en milieu hospitalier ‐ Le chemin parcouru par les bibliothécaires du CUSM. Congrès annuel de l'ABSC/CHLA; June 18, 2014; Montréal, Canada.

[14] Lyon JA , Garcia‐Milian R , Norton HF , Tennant MR . The use of research electronic data capture (REDCap) software to create a database of librarian‐mediated literature searches. Med Ref Serv Q. 2014;33(3):241‐252. 10.1080/02763869.2014.925379 25023012 PMC4339087

[15] SPSS Statistics [computer program]. Version 211989, 2012.

[16] Borah R , Brown AW , Capers PL , Kaiser KA . Analysis of the time and workers needed to conduct systematic reviews of medical interventions using data from the PROSPERO registry. BMJ Open. 2017;7(2):e012545 10.1136/bmjopen-2016-012545 PMC5337708 28242767

[17] Bullers K. Unpublished data (personal communication). 2017.

[18] Sampson M , Tetzlaff J , Urquhart C . Precision of healthcare systematic review searches in a cross‐sectional sample. Research Synthesis Methods. 2011;2(2):119‐125. 10.1002/jrsm.42 26061680

[19] Hausner E , Guddat C , Hermanns T , Lampert U , Waffenschmidt S . Development of search strategies for systematic reviews: validation showed the noninferiority of the objective approach. J Clin Epidemiol. 2015;68(2):191‐199. 10.1016/j.jclinepi.2014.09.016 25464826

[20] Pieterman K , Plaisier A , Govaert P , Leemans A , Lequin MH , Dudink J . Data quality in diffusion tensor imaging studies of the preterm brain: a systematic review. Pediatr Radiol. 2015;45(9):1372‐1381. 10.1007/s00247-015-3307-y 25820411 PMC4526590

[21] Bramer WM . Expert searching—searching for systematic reviews: set numbers or one liners?—column. MLA news. 2014;54(4):15

