# Supplementary material for: Evaluation of a new method for librarian‐mediated literature searches for systematic reviews
Source: Res Synth Methods. 2017 Nov 28;9(4):510–20. doi: 10.1002/jrsm.1279 (PMC5920798; doi:10.1002/jrsm.1279)
Supplement: Supplementary file 1 — Appendix Benchmark 1: Included ESM systematic reviews [file JRSM-9-510-s001.docx]

Appendix Benchmark 1 : Included ESM systematic reviews

| **Reference** | **# Boolean operators** | **# databases** | **# Deduplicated references** | **# included references** | **Precision** | **Search time (min)** |  |
| --- | --- | --- | --- | --- | --- | --- | --- |
| [Ahmadi, 2015 (1](#_ENREF_1)) | 95 | 6 | 2019 | 152 | 7,5% |  | acknowl. |
| [Ambagtsheer, 2016 (2](#_ENREF_2)) | 68 | 7 | 5216 | 86 | 1,6% |  | co-author |
| [Arabkhani, 2015 (3](#_ENREF_3)) | 10 | 7 | 2113 | 31 | 1,5% |  |  |
| [Atiq, 2015 (4](#_ENREF_4)) | 50 | 7 | 606 | 11 | 1,8% | 40 |  |
| [Baas, 2015 (5](#_ENREF_5)) | 29 | 7 | 427 | 14 | 3,3% | 60 |  |
| [Balak, 2016 (6](#_ENREF_6)) | 8 | 7 | 1058 | 68 | 6,4% | 30 | co-author |
| [Bijlard, 2015 (7](#_ENREF_7)) | 45 | 8 | 288 | 18 | 6,3% | 40 |  |
| [Blokker, 2016 (8](#_ENREF_8)) | 66 | 6 | 1506 | 16 | 1,1% |  | acknowl. |
| [Boersema, 2016 (9](#_ENREF_9)) | 73 | 6 | 4743 | 23 | 0,5% | 50 | acknowl. |
| [Buis, 2016 (10](#_ENREF_10)) | 39 | 7 | 1864 | 13 | 0,7% | 60 |  |
| [Chaker, 2014 (11](#_ENREF_11)) | 54 | 6 | 2273 | 6 | 0,3% |  | acknowl. |
| [Claessen, 2014 (12](#_ENREF_12)) | 28 | 7 | 985 | 22 | 2,2% | 40 | acknowl. |
| [Cnossen, 2015 (13](#_ENREF_13)) | 37 | 9 | 837 | 25 | 3,0% | 60 | acknowl. |
| [Dammeijer, 2016 (14](#_ENREF_14)) | 54 | 6 | 5629 | 18 | 0.3% | 70 | acknowl. |
| [De Bruijn, 2015 (15](#_ENREF_15)) | 26 | 6 | 1134 | 26 | 2,3% |  |  |
| [de Vos, 2014 (16](#_ENREF_16)) | 32 | 8 | 318 | 6 | 1,9% |  |  |
| [de Vos-Kerkhof, 2016 (17](#_ENREF_17)) | 88 | 7 | 2604 | 58 | 2,2% |  | acknowl. |
| [de Vries, 2015 (18](#_ENREF_18)) | 52 | 6 | 1119 | 14 | 1,3% |  | acknowl. |
| [Ekkelenkamp, 2016 (19](#_ENREF_19)) | 41 | 7 | 5846 | 94 | 1,6% |  |  |
| [Fischer, 2014 (20](#_ENREF_20)) | 22 | 6 | 2616 | 102 | 3,9% |  |  |
| [Garcia, 2016 (21](#_ENREF_21)) | 51 | 6 | 3967 | 81 | 2.0% |  | acknowl. |
| [Gerritsen, 2015 (22](#_ENREF_22)) | 50 | 3 | 5122 | 16 | 0,3% | 80 |  |
| [Harmeling, 2015 (23](#_ENREF_23)) | 50 | 7 | 1978 | 14 | 0,7% | 140 | acknowl. |
| [Hassing, 2015 (24](#_ENREF_24)) | 40 | 7 | 2156 | 11 | 0,5% | 90 |  |
| [Hogendoorn, 2014 (25](#_ENREF_25)) | 22 | 7 | 1490 | 47 | 3,2% |  |  |
| [Hosnijeh, 2015 (26](#_ENREF_26)) | 76 | 6 | 2411 | 25 | 1,0% | 90 |  |
| [Huygens, 2016 (27](#_ENREF_27)) | 59 | 4 | 1440 | 68 | 4.7% |  | acknowl. |
| [Ista, 2016 (28](#_ENREF_28)) | 64 | 6 | 2715 | 96 | 3,5% |  |  |
| [Jaspers, 2016 (29](#_ENREF_29)) | 18 | 13 | 285 | 5 | 1,8% | 100 | co-author |
| [Karim, 2016 (30](#_ENREF_30)) | 52 | 6 | 452 | 22 | 4.9% |  | co-author |
| [Kortram, 2014 (31](#_ENREF_31)) | 14 | 6 | 1030 | 21 | 2,0% |  | acknowl. |
| [Kortram, 2016 (32](#_ENREF_32)) | 24 | 7 | 2188 | 190 | 8.7% | 40 | acknowl. |
| [Kragt, 2015 (33](#_ENREF_33)) | 61 | 10 | 3837 | 40 | 1,0% |  | acknowl. |
| [Kroese, 2016 (34](#_ENREF_34)) | 32 | 8 | 401 | 10 | 2.5% | 80 | acknowl. |
| [Kuhlmann, 2016 (35](#_ENREF_35)) | 30 | 9 | 4452 | 10 | 0.2% |  | acknowl. |
| [Lafranca, 2015 (36](#_ENREF_36)) | 47 | 6 | 5526 | 56 | 1,0% |  | acknowl. |
| [le Clercq, 2016 (37](#_ENREF_37)) | 91 | 5 | 1050 | 33 | 3.1% | 60 |  |
| [Leermakers, 2015 (38](#_ENREF_38)) | 222 | 6 | 4386 | 50 | 1,1% |  | co-author |
| [Li, 2015 (39](#_ENREF_39)) | 120 | 6 | 340 | 13 | 3,8% | 50 | co-author |
| [Luk, 2015 (40](#_ENREF_40)) | 61 | 6 | 1663 | 62 | 3,7% | 90 | co-author |
| [Mookhoek, 2016 (41](#_ENREF_41)) | 23 | 6 | 982 | 46 | 4,7% | 100 |  |
| [Mosler, 2015 (42](#_ENREF_42)) | 29 | 9 | 2251 | 17 | 0,8% | 60 | acknowl. |
| [Muka, 2016 (43](#_ENREF_43)) | 70 | 6 | 9472 | 88 | 0,9% | 110 | co-author |
| [Muka, 2016 (44](#_ENREF_44)) | 57 | 6 | 3879 | 43 | 1.1% |  | co-author |
| [Muka, 2016 (45](#_ENREF_45)) | 48 | 11 | 3459 | 31 | 0.9% | 120 | co-author |
| [Nano, 2016 (46](#_ENREF_46)) | 35 | 6 | 4284 | 6 | 0.1% | 100 |  |
| [Pieterman, 2015 (47](#_ENREF_47)) | 27 | 7 | 2021 | 74 | 3.7% | 50 |  |
| [Pols, 2015 (48](#_ENREF_48)) | 88 | 5 | 1820 | 31 | 1,7% |  | acknowl. |
| [Rodenburg-Vlot, 2016 (49](#_ENREF_49)) | 73 | 8 | 2735 | 40 | 1,5% | 120 | acknowl. |
| [Roelants, 2015 (50](#_ENREF_50)) | 64 | 6 | 3901 | 16 | 0,4% |  |  |
| [Rokx, 2015 (51](#_ENREF_51)) | 45 | 7 | 994 | 19 | 1,9% |  | acknowl. |
| [Satoer, 2016 (52](#_ENREF_52)) | 87 | 10 | 3130 | 17 | 0,5% |  | acknowl. |
| [Scholten, 2016 (53](#_ENREF_53)) | 113 | 6 | 4800 | 68 | 1,4% | 100 |  |
| [Schoots, 2015 (54](#_ENREF_54)) | 45 | 8 | 3566 | 16 | 0,4% | 100 |  |
| [Serner, 2015 (55](#_ENREF_55)) | 69 | 9 | 2216 | 72 | 3,2% | 60 | acknowl. |
| [Strang, 2016 (56](#_ENREF_56)) | 24 | 7 | 3746 | 80 | 2.1% | 60 | acknowl. |
| [Suijkerbuijk, 2015 (57](#_ENREF_57)) | 56 | 6 | 2714 | 18 | 0,7% |  | acknowl. |
| [Suker, 2016 (58](#_ENREF_58)) | 13 | 7 | 728 | 13 | 1.8% | 30 |  |
| [Swart, 2016 (59](#_ENREF_59)) | 26 | 9 | 610 | 14 | 2.3% |  |  |
| [Taneri, 2016 (60](#_ENREF_60)) | 28 | 6 | 728 | 22 | 3.0% | 20 | co-author |
| [Ten Kate, 2015 (61](#_ENREF_61)) | 56 | 7 | 522 | 14 | 2,7% | 60 | acknowl. |
| [Tielemans, 2016 (62](#_ENREF_62)) | 39 | 8 | 7623 | 56 | 0,7% |  | co-author |
| [Tromp, 2016 (63](#_ENREF_63)) | 83 | 9 | 5245 | 38 | 0.7% |  | acknowl. |
| [van der Deijl, 2014 (64](#_ENREF_64)) | 67 | 7 | 2793 | 16 | 0.6% |  | acknowl. |
| [van der Does, 2016 (65](#_ENREF_65)) | 27 | 6 | 1492 | 9 | 0.6% | 50 | acknowl. |
| [van der Vaart, 2015 (66](#_ENREF_66)) | 30 | 6 | 5968 | 169 | 2,8% |  | acknowl. |
| [van Dijk, 2015 (67](#_ENREF_67)) | 25 | 7 | 2660 | 4 | 0,2% |  |  |
| [van Mol, 2015 (68](#_ENREF_68)) | 50 | 8 | 1620 | 40 | 2,5% |  | acknowl. |
| [Vargas, 2016 (69](#_ENREF_69)) | 79 | 6 | 3374 | 49 | 1.5% | 70 | co-author |
| [Voogt, 2015 (70](#_ENREF_70)) | 111 | 5 | 3811 | 14 | 0,4% |  |  |
| [Voortman, 2015 (71](#_ENREF_71)) | 232 | 3 | 2594 | 56 | 2,2% | 190 | co-author |
| [Wesseloo, 2016 (72](#_ENREF_72)) | 27 | 7 | 2090 | 37 | 1,8% |  |  |
| [Wu, 2016 (73](#_ENREF_73)) | 30 | 7 | 500 | 20 | 4.0% | 40 | acknowl. |
|  |  |  |  |  |  |  |  |
| *minimum* | 8 | 3 | 285 | 4 | 0.1% | 20 |  |
| *10 percentile* | 22 | 6 | 471 | 10 | 0.4% | 38 |  |
| *25 percentile* | 29 | 6 | 1040 | 14 | 0.7% | 50 |  |
| *median* | 50 | 7 | 2188 | 25 | 1.8% | 60 |  |
| *Average* | 54 | 6.9 | 2581 | 40 | 2.1% | 73 |  |
| *75 percentile* | 68 | 7 | 3824 | 56 | 3.0% | 100 |  |
| *90 percentile* | 90 | 9 | 5233 | 87 | 4.4% | 120 |  |
| *maximum* | 232 | 13 | 9472 | 190 | 8.7% | 190 |  |

1. Ahmadi AR, Lafranca JA, Claessens LA, Imamdi RM, JN IJ, Betjes MG, et al. Shifting paradigms in eligibility criteria for live kidney donation: a systematic review. Kidney Int. 2015 Jan;87(1):31-45. PubMed PMID: 24786706. Epub 2014/05/03. eng.

2. Ambagtsheer F, de Jong J, Bramer WM, Weimar W. On patients who purchase organ transplants abroad. American journal of transplantation : official journal of the American Society of Transplantation and the American Society of Transplant Surgeons. 2016 Mar 1. PubMed PMID: 26932422. Epub 2016/03/05. Eng.

3. Arabkhani B, Mookhoek A, Di Centa I, Lansac E, Bekkers JA, De Lind Van Wijngaarden R, et al. Reported Outcome After Valve-Sparing Aortic Root Replacement for Aortic Root Aneurysm: A Systematic Review and Meta-Analysis. The Annals of thoracic surgery. 2015 Sep;100(3):1126-31. PubMed PMID: 26228603. Epub 2015/08/01. eng.

4. Atiq F, van den Bemt PM, Leebeek FW, van Gelder T, Versmissen J. A systematic review on the accumulation of prophylactic dosages of low-molecular-weight heparins (LMWHs) in patients with renal insufficiency. European journal of clinical pharmacology. 2015 Aug;71(8):921-9. PubMed PMID: 26071276. Pubmed Central PMCID: PMC4500846. Epub 2015/06/14. eng.

5. Baas M, Duraku LS, Corten EM, Mureau MA. A systematic review on the sensory reinnervation of free flaps for tongue reconstruction: Does improved sensibility imply functional benefits? Journal of plastic, reconstructive & aesthetic surgery : JPRAS. 2015 Aug;68(8):1025-35. PubMed PMID: 26044088. Epub 2015/06/06. eng.

6. Balak DM, Fallah Arani S, Hajdarbegovic E, Hagemans CA, Bramer WM, Thio HB, et al. Efficacy, effectiveness, and safety of fumaric acid esters in the treatment of psoriasis: a systematic review of randomized and observational studies. Br J Dermatol. 2016 Feb 27. PubMed PMID: 26919824.

7. Bijlard E, Steltenpool S, Niessen FB. Intralesional 5-fluorouracil in keloid treatment: a systematic review. Acta dermato-venereologica. 2015 Sep;95(7):778-82. PubMed PMID: 25805099. Epub 2015/03/26. eng.

8. Blokker BM, Wagensveld IM, Weustink AC, Oosterhuis JW, Hunink MG. Non-invasive or minimally invasive autopsy compared to conventional autopsy of suspected natural deaths in adults: a systematic review. European radiology. 2016 Apr;26(4):1159-79. PubMed PMID: 26210206. Epub 2015/07/27. eng.

9. Boersema GS, Grotenhuis N, Bayon Y, Lange JF, Bastiaansen-Jenniskens YM. The Effect of Biomaterials Used for Tissue Regeneration Purposes on Polarization of Macrophages. BioResearch open access. 2016;5(1):6-14. PubMed PMID: 26862468. Pubmed Central PMCID: PMC4744891. Epub 2016/02/11. eng.

10. Buis ML, Maissan IM, Hoeks SE, Klimek M, Stolker RJ. Defining the learning curve for endotracheal intubation using direct laryngoscopy: A systematic review. Resuscitation. 2016 Feb;99:63-71. PubMed PMID: 26711127. Epub 2015/12/30. eng.

11. Chaker L, Baumgartner C, Ikram MA, Dehghan A, Medici M, Visser WE, et al. Subclinical thyroid dysfunction and the risk of stroke: a systematic review and meta-analysis. Eur J Epidemiol. 2014 Nov;29(11):791-800. PubMed PMID: 25179793. Epub 2014/09/03. eng.

12. Claessen FM, de Vos RJ, Reijman M, Meuffels DE. Predictors of primary Achilles tendon ruptures. Sports Med. 2014 Sep;44(9):1241-59. PubMed PMID: 24929701. Epub 2014/06/16. eng.

13. Cnossen MC, Scholten AC, Lingsma H, Synnot A, Tavender E, Gantner D, et al. Adherence to guidelines in adult patients with traumatic brain injury: A living systematic review. Journal of neurotrauma. 2015 Oct 2. PubMed PMID: 26431625. Epub 2015/10/04. Eng.

14. Dammeijer F, Lievense LA, Veerman GD, Hoogsteden HC, Hegmans JP, Arends LR, et al. The Efficacy of Tumor Vaccines and Cellular Immunotherapies in Non-Small Cell Lung Cancer: A Systematic Review and Meta-Analysis. Journal of clinical oncology : official journal of the American Society of Clinical Oncology. 2016 Jul 18. PubMed PMID: 27432922. Epub 2016/07/20. Eng.

15. De Bruijn KM, van Eijck CH. New-onset diabetes after distal pancreatectomy: a systematic review. Ann Surg. 2015 May;261(5):854-61. PubMed PMID: 24983994. Epub 2014/07/02. eng.

16. de Vos RJ, Windt J, Weir A. Strong evidence against platelet-rich plasma injections for chronic lateral epicondylar tendinopathy: a systematic review. Br J Sports Med. 2014 Jun;48(12):952-6. PubMed PMID: 24563387. Epub 2014/02/25. eng.

17. de Vos-Kerkhof E, Geurts DH, Wiggers M, Moll HA, Oostenbrink R. Tools for 'safety netting' in common paediatric illnesses: a systematic review in emergency care. Archives of disease in childhood. 2016 Feb;101(2):131-9. PubMed PMID: 26163122. Epub 2015/07/15. eng.

18. de Vries J, Ischebeck BK, Voogt LP, van der Geest JN, Janssen M, Frens MA, et al. Joint position sense error in people with neck pain: A systematic review. Manual therapy. 2015 Dec;20(6):736-44. PubMed PMID: 25983238. Epub 2015/05/20. eng.

19. Ekkelenkamp VE, Koch AD, de Man RA, Kuipers EJ. Training and competence assessment in GI endoscopy: a systematic review. Gut. 2016 Apr;65(4):607-15. PubMed PMID: 25636697. Epub 2015/02/01. eng.

20. Fischer C, Lingsma HF, Marang-van de Mheen PJ, Kringos DS, Klazinga NS, Steyerberg EW. Is the readmission rate a valid quality indicator? A review of the evidence. PLoS One. 2014;9(11):e112282. PubMed PMID: 25379675. Pubmed Central PMCID: PMC4224424. Epub 2014/11/08. eng.

21. Garcia AH, Voortman T, Baena CP, Chowdhurry R, Muka T, Jaspers L, et al. Maternal weight status, diet, and supplement use as determinants of breastfeeding and complementary feeding: a systematic review and meta-analysis. Nutr Rev. 2016 Jun 20. PubMed PMID: 27330143.

22. Gerritsen JK, Vincent AJ. Exercise improves quality of life in patients with cancer: a systematic review and meta-analysis of randomised controlled trials. Br J Sports Med. 2015 Dec 30. PubMed PMID: 26719503. Epub 2016/01/01. Eng.

23. Harmeling XJ, Kouwenberg CA, Bijlard E, Burger KN, Jager A, Mureau MA. The effect of immediate breast reconstruction on the timing of adjuvant chemotherapy: a systematic review. Breast cancer research and treatment. 2015 Sep;153(2):241-51. PubMed PMID: 26285643. Pubmed Central PMCID: PMC4559567. Epub 2015/08/20. eng.

24. Hassing RJ, Alsma J, Arcilla MS, van Genderen PJ, Stricker BH, Verbon A. International travel and acquisition of multidrug-resistant Enterobacteriaceae: a systematic review. Euro surveillance : bulletin Europeen sur les maladies transmissibles = European communicable disease bulletin. 2015 Nov 26;20(47). PubMed PMID: 26625301. Epub 2015/12/02. eng.

25. Hogendoorn W, Lavida A, Hunink MG, Moll FL, Geroulakos G, Muhs BE, et al. Open repair, endovascular repair, and conservative management of true splenic artery aneurysms. Journal of vascular surgery. 2014 Dec;60(6):1667-76 e1. PubMed PMID: 25264364. Epub 2014/09/30. eng.

26. Hosnijeh FS, Runhaar J, van Meurs JB, Bierma-Zeinstra SM. Biomarkers for osteoarthritis: Can they be used for risk assessment? A systematic review. Maturitas. 2015 Sep;82(1):36-49. PubMed PMID: 25963100. Epub 2015/05/13. eng.

27. Huygens SA, Mokhles MM, Hanif M, Bekkers JA, Bogers AJ, Rutten-van Molken MP, et al. Contemporary outcomes after surgical aortic valve replacement with bioprostheses and allografts: a systematic review and meta-analysis. European journal of cardio-thoracic surgery : official journal of the European Association for Cardio-thoracic Surgery. 2016 Mar 29. PubMed PMID: 27026750. Epub 2016/03/31. Eng.

28. Ista E, van der Hoven B, Kornelisse RF, van der Starre C, Vos MC, Boersma E, et al. Effectiveness of insertion and maintenance bundles to prevent central-line-associated bloodstream infections in critically ill patients of all ages: a systematic review and meta-analysis. The Lancet Infectious diseases. 2016 Feb 18. PubMed PMID: 26907734. Epub 2016/02/26. Eng.

29. Jaspers L, Feys F, Bramer WM, Franco OH, Leusink P, Laan ET. Efficacy and Safety of Flibanserin for the Treatment of Hypoactive Sexual Desire Disorder in Women: A Systematic Review and Meta-analysis. JAMA Intern Med. 2016 Apr 1;176(4):453-62. PubMed PMID: 26927498. Epub 2016/03/02. eng.

30. Karim F, Loeffen J, Bramer W, Westenberg L, Verdijk R, van Hagen M, et al. IgG4-related disease: a systematic review of this unrecognized disease in pediatrics. Pediatr Rheumatol Online J. 2016;14(1):18. PubMed PMID: 27012661. Pubmed Central PMCID: PMC4807566.

31. Kortram K, Lafranca JA, JN IJ, Dor FJ. The need for a standardized informed consent procedure in live donor nephrectomy: a systematic review. Transplantation. 2014 Dec 15;98(11):1134-43. PubMed PMID: 25436923. Epub 2014/12/02. eng.

32. Kortram K, Ijzermans JN, Dor FJ. Perioperative Events and Complications in Minimally Invasive Live Donor Nephrectomy: A Systematic Review and Meta-Analysis. Transplantation. 2016 Jul 15. PubMed PMID: 27428715. Epub 2016/07/19. Eng.

33. Kragt L, Dhamo B, Wolvius EB, Ongkosuwito EM. The impact of malocclusions on oral health-related quality of life in children-a systematic review and meta-analysis. Clinical oral investigations. 2015 Dec 4. PubMed PMID: 26635095. Epub 2015/12/05. Eng.

34. Kroese LF, de Smet GH, Jeekel J, Kleinrensink GJ, Lange JF. Systematic Review and Meta-Analysis of Extraperitoneal Versus Transperitoneal Colostomy for Preventing Parastomal Hernia. Diseases of the colon and rectum. 2016 Jul;59(7):688-95. PubMed PMID: 27270522. Epub 2016/06/09. eng.

35. Kuhlmann AY, Etnel JR, Roos-Hesselink JW, Jeekel J, Bogers AJ, Takkenberg JJ. Systematic review and meta-analysis of music interventions in hypertension treatment: a quest for answers. BMC cardiovascular disorders. 2016;16:69. PubMed PMID: 27095510. Pubmed Central PMCID: PMC4837643. Epub 2016/04/21. eng.

36. Lafranca JA, JN I, Betjes MG, Dor FJ. Body mass index and outcome in renal transplant recipients: a systematic review and meta-analysis. BMC Med. 2015;13:111. PubMed PMID: 25963131. Pubmed Central PMCID: PMC4427990. Epub 2015/05/13. eng.

37. le Clercq CM, van Ingen G, Ruytjens L, van der Schroeff MP. Music-induced Hearing Loss in Children, Adolescents, and Young Adults: A Systematic Review and Meta-analysis. Otology & neurotology : official publication of the American Otological Society, American Neurotology Society [and] European Academy of Otology and Neurotology. 2016 Jul 27. PubMed PMID: 27466893. Epub 2016/07/29. Eng.

38. Leermakers ET, Moreira EM, Kiefte-de Jong JC, Darweesh SK, Visser T, Voortman T, et al. Effects of choline on health across the life course: a systematic review. Nutr Rev. 2015 Aug;73(8):500-22. PubMed PMID: 26108618. Epub 2015/06/26. Eng.

39. Li J, Hernanda PY, Bramer WM, Peppelenbosch MP, van Luijk J, Pan Q. Anti-Tumor Effects of Metformin in Animal Models of Hepatocellular Carcinoma: A Systematic Review and Meta-Analysis. PLoS One. 2015;10(6):e0127967. PubMed PMID: 26030161. Epub 2015/06/02. Eng.

40. Luk F, de Witte SF, Bramer WM, Baan CC, Hoogduijn MJ. Efficacy of immunotherapy with mesenchymal stem cells in man: a systematic review. Expert review of clinical immunology. 2015 Mar 27;11(5):617-36. PubMed PMID: 25817052. Epub 2015/03/31. Eng.

41. Mookhoek A, Korteland NM, Arabkhani B, Di Centa I, Lansac E, Bekkers JA, et al. Bentall Procedure: A Systematic Review and Meta-Analysis. The Annals of thoracic surgery. 2016 Feb 5. PubMed PMID: 26857635. Epub 2016/02/10. Eng.

42. Mosler AB, Agricola R, Weir A, Holmich P, Crossley KM. Which factors differentiate athletes with hip/groin pain from those without? A systematic review with meta-analysis. Br J Sports Med. 2015 Jun;49(12):810. PubMed PMID: 26031646. Pubmed Central PMCID: PMC4484362. Epub 2015/06/03. eng.

43. Muka T, Vargas KG, Jaspers L, Wen KX, Dhana K, Vitezova A, et al. Estrogen receptor beta actions in the female cardiovascular system: A systematic review of animal and human studies. Maturitas. 2016 Apr;86:28-43. PubMed PMID: 26921926.

44. Muka T, Nano J, Voortman T, Braun KV, Ligthart S, Stranges S, et al. The role of global and regional DNA methylation and histone modifications in glycemic traits and type 2 diabetes: A systematic review. Nutr Metab Cardiovasc Dis. 2016 Jul;26(7):553-66. PubMed PMID: 27146363.

45. Muka T, Koromani F, Portilla E, O'Connor A, Bramer WM, Troup J, et al. The role of epigenetic modifications in cardiovascular disease: A systematic review. Int J Cardiol. 2016 Jun 1;212:174-83. PubMed PMID: 27038728. Epub 2016/04/04. eng.

46. Nano J, Muka T, Cepeda M, Voortman T, Dhana K, Brahimaj A, et al. Association of circulating total bilirubin with the metabolic syndrome and type 2 diabetes: A systematic review and meta-analysis of observational evidence. Diabetes & metabolism. 2016 Jul 5. PubMed PMID: 27396752. Epub 2016/07/12. Eng.

47. Pieterman K, Plaisier A, Govaert P, Leemans A, Lequin MH, Dudink J. Data quality in diffusion tensor imaging studies of the preterm brain: a systematic review. Pediatric radiology. 2015 Aug;45(9):1372-81. PubMed PMID: 25820411. Pubmed Central PMCID: PMC4526590. Epub 2015/03/31. eng.

48. Pols DH, Wartna JB, van Alphen EI, Moed H, Rasenberg N, Bindels PJ, et al. Interrelationships between Atopic Disorders in Children: A Meta-Analysis Based on ISAAC Questionnaires. PLoS One. 2015;10(7):e0131869. PubMed PMID: 26135565. Pubmed Central PMCID: PMC4489894. Epub 2015/07/03. eng.

49. Rodenburg-Vlot MB, Ruytjens L, Oostenbrink R, Goedegebure A, van der Schroeff MP. Systematic Review: Incidence and Course of Hearing Loss Caused by Bacterial Meningitis: In Search of an Optimal Timed Audiological Follow-up. Otology & neurotology : official publication of the American Otological Society, American Neurotology Society [and] European Academy of Otology and Neurotology. 2016 Jan;37(1):1-8. PubMed PMID: 26649601. Epub 2015/12/10. Eng.

50. Roelants JA, de Jonge RC, Steegers-Theunissen RP, Reiss IK, Joosten KF, Vermeulen MJ. Prenatal markers of neonatal fat mass: A systematic review. Clinical nutrition (Edinburgh, Scotland). 2015 Sep 25. PubMed PMID: 26499032. Epub 2015/10/27. Eng.

51. Rokx C, Rijnders BJ, van Laar JA. Treatment of multicentric Castleman&rsquo;s disease in HIV-1 infected and uninfected patients: a systematic review. The Netherlands journal of medicine. 2015 Jun;73(5):202-10. PubMed PMID: 26087799. Epub 2015/06/20. eng.

52. Satoer D, Visch-Brink E, Dirven C, Vincent A. Glioma surgery in eloquent areas: can we preserve cognition? Acta neurochirurgica. 2016 Jan;158(1):35-50. PubMed PMID: 26566782. Pubmed Central PMCID: PMC4684586. Epub 2015/11/15. eng.

53. Scholten AC, Haagsma JA, Cnossen MC, Olff M, Van Beeck EF, Polinder S. Prevalence and risk factors of anxiety and depressive disorders following traumatic brain injury: a systematic review. Journal of neurotrauma. 2016 Jan 5. PubMed PMID: 26729611. Epub 2016/01/06. Eng.

54. Schoots IG, Roobol MJ, Nieboer D, Bangma CH, Steyerberg EW, Hunink MG. Magnetic resonance imaging-targeted biopsy may enhance the diagnostic accuracy of significant prostate cancer detection compared to standard transrectal ultrasound-guided biopsy: a systematic review and meta-analysis. Eur Urol. 2015 Sep;68(3):438-50. PubMed PMID: 25480312. Epub 2014/12/07. eng.

55. Serner A, van Eijck CH, Beumer BR, Holmich P, Weir A, de Vos RJ. Study quality on groin injury management remains low: a systematic review on treatment of groin pain in athletes. Br J Sports Med. 2015 Jun;49(12):813. PubMed PMID: 25633830. Pubmed Central PMCID: PMC4484372. Epub 2015/01/31. eng.

56. Strang SG, Van Lieshout EM, Van Waes OJ, Verhofstad MH. Prevalence and mortality of abdominal compartment syndrome in severely injured patients; A systematic review. The journal of trauma and acute care surgery. 2016 Jul 8. PubMed PMID: 27398983. Epub 2016/07/12. Eng.

57. Suijkerbuijk MA, Reijman M, Lodewijks SJ, Punt J, Meuffels DE. Hamstring Tendon Regeneration After Harvesting: A Systematic Review. Am J Sports Med. 2015 Oct;43(10):2591-8. PubMed PMID: 25548149. Epub 2014/12/31. eng.

58. Suker M, Beumer BR, Sadot E, Marthey L, Faris JE, Mellon EA, et al. FOLFIRINOX for locally advanced pancreatic cancer: a systematic review and patient-level meta-analysis. The Lancet Oncology. 2016 Jun;17(6):801-10. PubMed PMID: 27160474. Epub 2016/05/11. eng.

59. Swart NM, van Oudenaarde K, Reijnierse M, Nelissen RG, Verhaar JA, Bierma-Zeinstra SM, et al. Effectiveness of exercise therapy for meniscal lesions in adults: A systematic review and meta-analysis. Journal of science and medicine in sport / Sports Medicine Australia. 2016 Apr 20. PubMed PMID: 27129638. Epub 2016/05/01. Eng.

60. Taneri PE, Kiefte-de Jong JC, Bramer WM, Daan NM, Franco OH, Muka T. Association of alcohol consumption with the onset of natural menopause: a systematic review and meta-analysis. Human reproduction update. 2016 Jun 8. PubMed PMID: 27278232. Epub 2016/06/10. Eng.

61. Ten Kate CA, Tibboel D, Kraemer US. B-type natriuretic peptide as a parameter for pulmonary hypertension in children. A systematic review. European journal of pediatrics. 2015 Oct;174(10):1267-75. PubMed PMID: 26298682. Epub 2015/08/25. eng.

62. Tielemans MJ, Garcia AH, Peralta Santos A, Bramer WM, Luksa N, Luvizotto MJ, et al. Macronutrient composition and gestational weight gain: a systematic review. The American journal of clinical nutrition. 2016 Jan;103(1):83-99. PubMed PMID: 26675773. Epub 2015/12/18. eng.

63. Tromp K, Zwaan CM, van de Vathorst S. Motivations of children and their parents to participate in drug research: a systematic review. European journal of pediatrics. 2016 May;175(5):599-612. PubMed PMID: 27041121. Pubmed Central PMCID: PMC4839044. Epub 2016/04/05. eng.

64. van der Deijl M, Etman A, Kamphuis CB, van Lenthe FJ. Participation levels of physical activity programs for community-dwelling older adults: a systematic review. BMC Public Health. 2014;14:1301. PubMed PMID: 25523712. Pubmed Central PMCID: PMC4301079. Epub 2014/12/20. eng.

65. van der Does Y, Rood PP, Haagsma JA, Patka P, van Gorp EC, Limper M. Procalcitonin-guided therapy for the initiation of antibiotics in the ED: a systematic review. The American journal of emergency medicine. 2016 Jul;34(7):1286-93. PubMed PMID: 27130585. Epub 2016/05/01. eng.

66. van der Vaart T, Overwater IE, Oostenbrink R, Moll HA, Elgersma Y. Treatment of Cognitive Deficits in Genetic Disorders: A Systematic Review of Clinical Trials of Diet and Drug Treatments. JAMA neurology. 2015 Sep;72(9):1052-60. PubMed PMID: 26168015. Epub 2015/07/15. eng.

67. van Dijk GM, Maneva M, Colpani V, Dhana K, Muka T, Jaspers L, et al. The association between vasomotor symptoms and metabolic health in peri- and postmenopausal women: a systematic review. Maturitas. 2015 Feb;80(2):140-7. PubMed PMID: 25532993. Epub 2014/12/24. eng.

68. van Mol MM, Kompanje EJ, Benoit DD, Bakker J, Nijkamp MD. The Prevalence of Compassion Fatigue and Burnout among Healthcare Professionals in Intensive Care Units: A Systematic Review. PLoS One. 2015;10(8):e0136955. PubMed PMID: 26322644. Pubmed Central PMCID: PMC4554995. Epub 2015/09/01. eng.

69. Vargas KG, Milic J, Zaciragic A, Wen KX, Jaspers L, Nano J, et al. The functions of estrogen receptor beta in the female brain: A systematic review. Maturitas. 2016 Jun 4. PubMed PMID: 27338976. Epub 2016/06/25. Eng.

70. Voogt L, de Vries J, Meeus M, Struyf F, Meuffels D, Nijs J. Analgesic effects of manual therapy in patients with musculoskeletal pain: a systematic review. Manual therapy. 2015 Apr;20(2):250-6. PubMed PMID: 25282440. Epub 2014/10/06. eng.

71. Voortman T, van den Hooven EH, Braun KV, van den Broek M, Bramer WM, Chowdhurry R, et al. Effects of polyunsaturated fatty acid intake and status during pregnancy, lactation, and early childhood on cardiometabolic health: A systematic review. Prog Lipid Res. 2015 May 27;59:67-87. PubMed PMID: 26025302. Epub 2015/05/31. Eng.

72. Wesseloo R, Kamperman AM, Munk-Olsen T, Pop VJ, Kushner SA, Bergink V. Risk of Postpartum Relapse in Bipolar Disorder and Postpartum Psychosis: A Systematic Review and Meta-Analysis. The American journal of psychiatry. 2016 Feb 1;173(2):117-27. PubMed PMID: 26514657. Epub 2015/10/31. eng.

73. Wu Z, van de Haar RC, Sparreboom CL, Boersema GS, Li Z, Ji J, et al. Is the intraoperative air leak test effective in the prevention of colorectal anastomotic leakage? A systematic review and meta-analysis. International journal of colorectal disease. 2016 Aug;31(8):1409-17. PubMed PMID: 27294661. Pubmed Central PMCID: PMC4947486. Epub 2016/06/14. eng.
