# Supplementary material for: Evaluation of a new method for librarian‐mediated literature searches for systematic reviews
Source: Res Synth Methods. 2017 Nov 28;9(4):510–20. doi: 10.1002/jrsm.1279 (PMC5920798; doi:10.1002/jrsm.1279)
Supplement: Supplementary file 2 — Appendix Benchmark 2: Included DAH systematic reviews [file JRSM-9-510-s002.docx]

Appendix Benchmark 2 : Included DAH systematic reviews

|  | **# Search terms** | **# databases** | **# Deduplicated references** | **# included references** | **Precision** | **institute** | **Co-author** |
| --- | --- | --- | --- | --- | --- | --- | --- |
| [Abrahams 2016 (1)](#_ENREF_1) | 52 | 5 | 3392 | 27 | 0.8% | nijmegen |  |
| [Akintola 2015 (2)](#_ENREF_2) |  | 3 | 1384 | 8 | 0.6% | amc |  |
| [Alam 2014 (3)](#_ENREF_3) |  | 1 | 788 | 2 | 0.3% | vu |  |
| [Andela 2015 (4)](#_ENREF_4) | 78 | 2 | 6699 | 43 | 0.6% | amc |  |
| [Baltes 2016 (5)](#_ENREF_5) | 28 | 3 | 1090 | 23 | 2.1% | amc |  |
| [Batelaan 2016 (6)](#_ENREF_6) | 49 | 3 | 2489 | 67 | 2.7% | vu |  |
| [Beeldman 2016 (7)](#_ENREF_7) | 54 | 4 | 312 | 6 | 1.9% | amc | co-author |
| [Beishuizen 2016 (8)](#_ENREF_8) | 147 | 4 | 5251 | 84 | 1.6% | amc |  |
| [Bij de Vaate 2014 (9)](#_ENREF_9) | 28 | 2 | 2957 | 21 | 0.7% | vu |  |
| [Bijker 2016 (10)](#_ENREF_10) | 250 | 3 | 2391 | 163 | 6.8% | amc |  |
| [Bittermann 2014 (11)](#_ENREF_11) | 34 | 4 | 1350 | 15 | 1.1% | utrecht |  |
| [Bleeker 2015 (12)](#_ENREF_12) | 123 | 3 | 724 |  |  | amc |  |
| [Bleker 2016 (13)](#_ENREF_13) | 56 | 3 | 4267 | 45 | 1.1% | amc | co-author |
| [Blikkendaal 2015 (14)](#_ENREF_14) | 134 | 8 | 3616 | 74 | 2.0% | leiden |  |
| [Boender 2015 (15)](#_ENREF_15) | 30 | 2 | 617 | 30 | 4.9% | amc |  |
| [Boerebach 2014 (16)](#_ENREF_16) | 39 | 3 | 373 | 5 | 1.3% | utrecht |  |
| [Boom 2015 (17)](#_ENREF_17) |  | 5 | 4050 | 12 | 0.3% | amc |  |
| [Borgert 2015 (18)](#_ENREF_18) | 55 | 3 | 1307 | 26 | 2.0% | amc |  |
| [Borsje 2015 (19)](#_ENREF_19) |  | 3 | 1115 | 5 |  | amc |  |
| [Breugom 2015 (20)](#_ENREF_20) |  | 2 | 3254 | 39 | 1.2% | leiden |  |
| [Broeder 2014 (21)](#_ENREF_21) | 79 | 3 | 13382 | 3 | 0.0% | utrecht |  |
| [Broersen 2015 (22)](#_ENREF_22) | 397 | 2 | 4527 | 1342 | 29.6% | nijmegen |  |
| [Brom 2014 (23)](#_ENREF_23) |  | 5 | 2297 | 144 | 6.3% | nijmegen |  |
| [Brons 2014 (24)](#_ENREF_24) |  | 3 | 681 | 26 | 3.8% | nijmegen |  |
| [Bruijnzeel 2016 (25)](#_ENREF_25) |  | 3 | 3353 | 10 | 0.3% | utrecht |  |
| [Bruijnzeel 2016 (26)](#_ENREF_26) | 26 | 2 | 294 | 4 | 1.4% | utrecht |  |
| [Bruijnzeel 2016 (27)](#_ENREF_27) | 23 | 2 | 74 | 44 | 59.5% | utrecht |  |
| [Brunsveld-Reinders 2016 (28)](#_ENREF_28) |  | 3 | 2132 | 62 | 2.9% | amc |  |
| [Burger 2015 (29)](#_ENREF_29) | 28 | 2 | 648 | 30 | 4.6% | amc |  |
| [Busard 2014 (30)](#_ENREF_30) | 68 | 8 | 429 | 140 | 32.6% | amc |  |
| [Charehbili 2014 (31)](#_ENREF_31) | 43 | 1 | 228 | 26 | 11.4% | leiden |  |
| [Coelen 2016 (32)](#_ENREF_32) | 18 | 2 | 173 | 12 | 6.9% | amc |  |
| [Conijn 2015 (33)](#_ENREF_33) | 61 | 3 | 2876 | 20 | 0.7% | vu |  |
| [Danhof 2015 (34)](#_ENREF_34) | 31 | 2 | 1594 | 22 | 1.4% | leiden |  |
| [de Glas 2015 (35)](#_ENREF_35) | 49 | 4 | 1364 | 102 | 7.5% | leiden |  |
| [de Groof 2016 (36)](#_ENREF_36) | 32 | 3 | 313 | 10 | 3.2% | amc |  |
| [de Groof 2016 (37)](#_ENREF_37) | 31 | 3 | 384 | 12 | 3.1% | amc |  |
| [de Hundt 2014 (38)](#_ENREF_38) |  | 2 | 1246 | 40 | 3.2% | amc |  |
| [de Jong 2014 (39)](#_ENREF_39) | 13 | 3 | 366 | 13 | 3.6% | leiden |  |
| [de Ruiter 2014 (40)](#_ENREF_40) | 97 | 3 | 1659 | 26 | 1.6% | amc |  |
| [de Vries 2014 (41)](#_ENREF_41) |  | 4 | 1626 | 33 | 2.0% | utrecht |  |
| [de Vries 2015 (42)](#_ENREF_42) | 6 | 3 | 1046 | 11 | 1.1% | vu | co-author |
| [de Vries 2016 (43)](#_ENREF_43) |  | 3 | 3764 | 66 | 1.8% | utrecht |  |
| [de Wit 2016 (44)](#_ENREF_44) |  | 3 | 907 | 67 | 7.4% | nijmegen |  |
| [Delli 2015 (45)](#_ENREF_45) | 40 | 5 | 2951 | 58 | 2.0% | amc |  |
| [Demirdas 2015 (46)](#_ENREF_46) | 325 | 4 | 620 | 20 | 3.2% | amc | co-author |
| [Dietz 2015 (47)](#_ENREF_47) | 39 | 3 | 1307 | 22 | 1.7% | vu |  |
| [Donker-Cools 2016 (48)](#_ENREF_48) | 27 | 3 | 1575 | 15 | 1.0% | utrecht |  |
| [Driessen 2015 (49)](#_ENREF_49) | 56 | 3 | 827 | 10 | 1.2% | utrecht |  |
| [Driessen 2016 (50)](#_ENREF_50) |  | 3 | 487 | 27 | 5.5% | utrecht |  |
| [Dubois 2015 (51)](#_ENREF_51) | 46 | 6 | 1535 | 22 | 1.4% | groningen |  |
| [Dubois 2015 (52)](#_ENREF_52) | 59 | 1 | 792 | 17 | 2.1% | amc |  |
| [Ebisch 2016 (53)](#_ENREF_53) | 44 | 1 | 491 | 63 | 12.8% | leiden |  |
| [Engelhardt 2014 (54)](#_ENREF_54) | 59 | 3 | 996 | 26 | 2.6% | leiden |  |
| [Flik 2015 (55)](#_ENREF_55) | 24 | 3 | 2866 | 50 | 1.7% | amc |  |
| [Font-Gonzalez 2016 (56)](#_ENREF_56) | 194 | 1 | 1074 | 11 | 1.0% | amc |  |
| [Fransen 2016 (57)](#_ENREF_57) |  | 1 | 1373 | 13 | 0.9% | nijmegen |  |
| [Frerichs 2014 (58)](#_ENREF_58) | 127 | 3 | 13202 | 2 | 0.0% | utrecht |  |
| [Garritsen 2014 (59)](#_ENREF_59) | 190 | 6 | 1066 | 19 | 1.8% | amc | co-author |
| [Geboers 2015 (60)](#_ENREF_60) | 33 | 5 | 3118 | 9 | 0.3% | amc |  |
| [Gerbens 2016 (61)](#_ENREF_61) | 455 | 3 | 2332 | 29 | 1.2% | amc | co-author |
| [Gielen 2014 (62)](#_ENREF_62) |  | 7 | 959 | 20 | 2.1% | leiden | co-author |
| [Glaudemans 2015 (63)](#_ENREF_63) |  | 2 | 1008 | 37 | 3.7% | nijmegen |  |
| [Gorter 2014 (64)](#_ENREF_64) | 77 | 3 | 1350 | 55 | 4.1% | amc |  |
| [Govaert 2015 (65)](#_ENREF_65) | 23 | 4 | 232 | 41 |  | leiden |  |
| [Groenen 2016 (66)](#_ENREF_66) | 173 | 3 | 3273 | 39 | 1.2% | nijmegen |  |
| [Grooten 2015 (67)](#_ENREF_67) | 30 | 2 | 119 | 5 | 4.2% | amc |  |
| [Gvozdenovic 2014 (68)](#_ENREF_68) | 558 | 3 | 6557 | 13 | 0.2% | amc | co-author |
| [Hamelinck 2014 (69)](#_ENREF_69) |  | 4 | 9012 | 18 | 0.2% | vu |  |
| [Harmsen 2015 (70)](#_ENREF_70) | 80 | 3 | 6108 |  |  | amc |  |
| [Harmsen 2016 (71)](#_ENREF_71) | 60 | 3 | 186 | 12 | 6.5% | vu | co-author |
| [Haroun 2016 (72)](#_ENREF_72) | 41 | 4 | 1351 | 18 | 1.3% | amc | co-author |
| [Hassell 2015 (73)](#_ENREF_73) |  | 7 | 300 | 26 | 8.7% | leiden |  |
| [Hauer 2014 (74)](#_ENREF_74) | 162 | 3 | 3171 | 1 | 0.0% | utrecht |  |
| [Havermans 2016 (75)](#_ENREF_75) |  | 7 | 3613 | 44 | 1.2% | vu |  |
| [Heida 2016 (76)](#_ENREF_76) | 52 | 4 | 3608 | 6 | 0.2% | leiden |  |
| [Heitink-Polle 2014 (77)](#_ENREF_77) | 90 | 4 | 751 | 13 | 1.7% | leiden |  |
| [Helmerhorst 2015 (78)](#_ENREF_78) |  | 5 | 6186 | 8 | 0.1% | utrecht |  |
| [Hentschel 2014 (79)](#_ENREF_79) | 86 | 3 | 2038 | 119 | 5.8% | leiden |  |
| [Hoencamp 2014 (80)](#_ENREF_80) | 92 | 4 | 2168 | 17 | 0.8% | amc |  |
| [Hofstede 2015 (81)](#_ENREF_81) | 116 | 1 | 7722 | 77 | 1.0% | utrecht |  |
| [Hofstede 2016 (82)](#_ENREF_82) |  | 7 | 2595 | 35 | 1.3% | leiden |  |
| [Hoogedoorn 2015 (83)](#_ENREF_83) | 10 | 2 | 780 |  |  | nijmegen |  |
| [Hooker 2016 (84)](#_ENREF_84) |  | 3 | 372 | 2 | 0.5% | vu |  |
| [Horbach 2016 (85)](#_ENREF_85) |  | 4 | 17956 | 26 | 0.1% | vu |  |
| [Huisman 2016 (86)](#_ENREF_86) | 41 | 2 | 3792 | 9 | 0.2% | groningen |  |
| [Ingelse 2016 (87)](#_ENREF_87) |  | 3 | 2757 | 5 | 0.2% | amc | co-author |
| [Inklaar 2014 (88)](#_ENREF_88) |  | 8 | 242 | 11 | 4.5% | leiden |  |
| [Jalalzadeh 2016 (89)](#_ENREF_89) | 72 | 3 | 1933 | 7 | 0.4% | amc |  |
| [Jansen 2015 (90)](#_ENREF_90) | 93 | 8 | 2609 | 20 | 0.8% | leiden |  |
| [Jansen 2016 (91)](#_ENREF_91) | 33 | 5 | 3030 | 8 | 0.3% | amc |  |
| [Jens 2014 (92)](#_ENREF_92) | 54 | 3 | 2716 | 12 | 0.4% | leiden |  |
| [Jilesen 2016 (93)](#_ENREF_93) | 58 | 4 | 572 | 13 | 2.3% | amc | co-author |
| [Jonker 2016 (94)](#_ENREF_94) | 33 | 2 | 300 | 35 | 11.7% | nijmegen |  |
| [Kabaghe 2016 (95)](#_ENREF_95) | 57 | 1 | 474 | 14 | 3.0% | amc | co-author |
| [Kadouch 2015 (96)](#_ENREF_96) |  | 2 | 3546 | 36 | 1.0% | vu | co-author |
| [Kamalski 2014 (97)](#_ENREF_97) | 27 | 3 | 1448 | 8 | 0.6% | utrecht |  |
| [Kampshoff 2014 (98)](#_ENREF_98) | 19 | 5 | 1001 | 14 | 1.4% | nijmegen |  |
| [Kaptein 2015 (99)](#_ENREF_99) | 128 | 7 | 270 | 15 | 5.6% | leiden | co-author |
| [Kazemier 2014 (100)](#_ENREF_100) | 49 | 4 | 535 | 7 | 1.3% | leiden |  |
| [Kernkamp 2016 (101)](#_ENREF_101) | 36 | 8 | 866 | 6 | 0.7% | leiden | co-author |
| [Keurentjes 2014 (102)](#_ENREF_102) | 98 | 1 | 1048 | 232 | 22.1% | amc |  |
| [Khorsand 2015 (103)](#_ENREF_103) | 85 | 4 | 583 | 8 | 1.4% | amc | co-author |
| [Klaassen 2014 (104)](#_ENREF_104) | 15 | 3 | 571 | 10 | 1.8% | nijmegen |  |
| [Klein Hesselink 2015 (105)](#_ENREF_105) |  | 6 | 1131 | 4 | 0.4% | leiden |  |
| [Kluitenberg 2015 (106)](#_ENREF_106) | 82 | 3 | 1609 | 24 | 1.5% | leiden |  |
| [Kodde 2016 (107)](#_ENREF_107) | 20 | 3 | 264 | 27 | 10.2% | amc |  |
| [Korevaar 2014 (108)](#_ENREF_108) |  | 6 | 7195 | 467 | 6.5% | vu |  |
| [Korevaar 2014 (109)](#_ENREF_109) | 21 | 3 | 1496 | 16 | 1.1% | amc | co-author |
| [Korterink 2015 (110)](#_ENREF_110) |  | 4 | 652 | 20 | 3.1% | vu |  |
| [Kroon 2016 (111)](#_ENREF_111) | 49 | 20 | 2873 | 36 | 1.3% | leiden | co-author |
| [Kuijpers 2014 (112)](#_ENREF_112) | 232 | 5 | 415 | 19 | 4.6% | amc | co-author |
| [Kuizenga-Wessel 2015 (113)](#_ENREF_113) | 49 | 2 | 1232 | 57 | 4.6% | leiden |  |
| [Kuizenga-Wessel 2016 (114)](#_ENREF_114) | 91 | 3 | 3982 | 45 | 1.1% | amc |  |
| [la Chapelle 2015 (115)](#_ENREF_115) |  | 6 | 1091 | 22 | 2.0% | leiden |  |
| [Labots 2014 (116)](#_ENREF_116) | 421 | 4 | 2583 | 17 | 0.7% | amc |  |
| [Lambers Heerspink 2014 (117)](#_ENREF_117) | 25 | 3 | 669 | 7 | 1.0% | amc |  |
| [Lantinga 2015 (118)](#_ENREF_118) | 34 | 4 | 5513 | 62 | 1.1% | leiden | co-author |
| [Lemmers 2016 (119)](#_ENREF_119) |  | 3 | 373 | 8 | 2.1% | amc |  |
| [Lo-Fo-Wong 2015 (120)](#_ENREF_120) | 51 | 2 | 1293 | 19 | 1.5% | amc |  |
| [Mallee 2014 (121)](#_ENREF_121) | 10 | 3 | 644 | 81 | 12.6% | vu |  |
| [Mallee 2015 (122)](#_ENREF_122) | 20 | 3 | 2476 | 6 | 0.2% | nijmegen |  |
| [Man 2016 (123)](#_ENREF_123) | 30 | 4 | 293 | 11 | 3.8% | leiden |  |
| [Meiboom 2015 (124)](#_ENREF_124) | 48 | 3 | 2900 | 11 | 0.4% | amc |  |
| [Meijer 2014 (125)](#_ENREF_125) | 22 | 3 | 955 | 14 | 1.5% | utrecht |  |
| [Menting 2016 (126)](#_ENREF_126) |  | 8 | 1089 | 8 | 0.7% | leiden |  |
| [Metsaars 2014 (127)](#_ENREF_127) |  | 3 | 1959 | 6 | 0.3% | amc |  |
| [Meuleman 2015 (128)](#_ENREF_128) | 39 | 4 | 2597 | 6 | 0.2% | amc |  |
| [Mulder 2014 (129)](#_ENREF_129) |  | 3 | 10476 | 85 | 0.8% | amc |  |
| [Mulder 2015 (130)](#_ENREF_130) | 101 | 4 | 1350 | 21 | 1.6% | vu |  |
| [Muntingh 2016 (131)](#_ENREF_131) |  | 5 | 3073 | 7 | 0.2% | vu |  |
| [Nauta 2014 (132)](#_ENREF_132) |  | 5 | 5377 | 11 | 0.2% | vu |  |
| [Navarro-Compan 2015 (133)](#_ENREF_133) | 17 | 3 | 2313 | 35 | 1.5% | utrecht |  |
| [Negenborn 2016 (134)](#_ENREF_134) | 40 | 4 | 4801 | 286 | 6.0% | vu | co-author |
| [Niemeijer 2014 (135)](#_ENREF_135) | 424 | 2 |  |  |  | amc |  |
| [Ochodo 2014 (136)](#_ENREF_136) | 26 | 3 | 879 | 20 | 2.3% | leiden |  |
| [Ochodo 2015 (137)](#_ENREF_137) | 9 | 2 | 340 | 23 | 6.8% | amc |  |
| [Olde Engberink 2015 (138)](#_ENREF_138) |  | 5 | 13085 | 85 | 0.6% | amc |  |
| [Olthof 2014 (139)](#_ENREF_139) | 40 | 3 | 317 | 10 | 3.2% | groningen |  |
| [Onrust 2016 (140)](#_ENREF_140) |  | 4 | 3635 | 9 | 0.2% | amc |  |
| [Overdevest 2015 (141)](#_ENREF_141) | 93 | 3 | 3501 | 7 | 0.2% | utrecht |  |
| [Owusu 2015 (142)](#_ENREF_142) |  | 4 | 6885 | 77 | 1.1% | nijmegen |  |
| [Paap 2014 (143)](#_ENREF_143) |  | 3 | 7685 | 51 | 0.7% | amc |  |
| [Peeters 2014 (144)](#_ENREF_144) |  | 2 | 1158 | 21 | 1.8% | utrecht |  |
| [Peters 2016 (145)](#_ENREF_145) | 63 | 2 | 551 | 38 | 6.9% | vu |  |
| [Pijls 2016 (146)](#_ENREF_146) | 226 | 10 | 937 | 47 | 5.0% | leiden | co-author |
| [Proper 2016 (147)](#_ENREF_147) | 184 | 4 | 367 | 39 | 10.6% | vu |  |
| [Prosman 2015 (148)](#_ENREF_148) |  | 4 | 191 | 4 | 2.1% | amc |  |
| [Rashaan 2014 (149)](#_ENREF_149) | 31 | 3 | 1806 | 23 | 1.3% | vu |  |
| [Rashid 2016 (150)](#_ENREF_150) | 65 | 7 | 1963 | 17 | 0.9% | groningen |  |
| [Reinink 2014 (151)](#_ENREF_151) | 30 | 5 | 338 | 22 | 6.5% | leiden |  |
| [Roach 2015 (152)](#_ENREF_152) | 64 | 7 | 91 | 12 | 13.2% | leiden | co-author |
| [Roekevisch 2014 (153)](#_ENREF_153) | 238 | 3 | 465 | 43 | 9.2% | amc |  |
| [Rostamian 2014 (154)](#_ENREF_154) | 31 | 5 | 383 | 2 | 0.5% | utrecht |  |
| [Ruiter 2015 (155)](#_ENREF_155) | 73 | 3 | 5590 | 41 | 0.7% | nijmegen |  |
| [Ruiter 2016 (156)](#_ENREF_156) | 16 | 7 | 232 | 2 | 0.9% | utrecht |  |
| [Ruys 2014 (157)](#_ENREF_157) | 8 | 2 | 1789 | 73 | 4.1% | amc |  |
| [San Giorgi 2016 (158)](#_ENREF_158) | 21 | 3 | 1277 | 19 | 1.5% | groningen |  |
| [Sarac 2015 (159)](#_ENREF_159) | 45 | 2 | 4693 | 11 | 0.2% | amc |  |
| [Schaafsma 2016 (160)](#_ENREF_160) | 32 | 7 | 695 | 25 | 3.6% | utrecht |  |
| [Scheper 2016 (161)](#_ENREF_161) | 22 | 2 |  | 16 |  | amc |  |
| [Schimmer 2016 (162)](#_ENREF_162) | 25 | 2 | 824 | 12 | 1.5% | vu |  |
| [Schipper 2016 (163)](#_ENREF_163) | 19 | 4 | 3753 | 21 | 0.6% | vu |  |
| [Schopman 2014 (164)](#_ENREF_164) | 44 | 2 | 2338 | 25 | 1.1% | amc |  |
| [Schrijver 2016 (165)](#_ENREF_165) | 50 | 4 | 508 | 21 | 4.1% | amc |  |
| [Schuts 2016 (166)](#_ENREF_166) | 58 | 3 | 16387 | 146 | 0.9% | amc |  |
| [Sloothaak 2014 (167)](#_ENREF_167) | 20 | 4 | 479 | 5 | 1.0% | amc |  |
| [Smeets 2016 (168)](#_ENREF_168) | 11 | 2 | 873 | 6 | 0.7% | nijmegen |  |
| [Smeulers 2015 (169)](#_ENREF_169) |  |  |  | 80 |  | leiden | co-author |
| [Smits 2014 (170)](#_ENREF_170) | 51 | 4 | 2072 | 13 | 0.6% | amc |  |
| [Steenen 2016 (171)](#_ENREF_171) | 234 | 2 | 2110 | 21 | 1.0% | amc | co-author |
| [Steutel 2014 (172)](#_ENREF_172) |  | 1 |  | 26 |  | amc |  |
| [Stoekenbroek 2014 (173)](#_ENREF_173) | 29 | 2 | 3769 | 32 | 0.8% | amc |  |
| [Storm-Versloot 2014 (174)](#_ENREF_174) | 46 | 5 |  | 15 |  | amc |  |
| [Straatman 2015 (175)](#_ENREF_175) | 41 | 3 | 2511 | 13 | 0.5% | nijmegen |  |
| [Sutterland 2015 (176)](#_ENREF_176) | 73 | 3 | 1682 | 5 | 0.3% | amc |  |
| [Teepen 2016 (177)](#_ENREF_177) | 264 | 2 | 1969 | 45 | 2.3% | amc |  |
| [Terpstra 2014 (178)](#_ENREF_178) | 18 | 1 | 509 | 54 | 10.6% | vu |  |
| [Theunissen 2014 (179)](#_ENREF_179) | 142 | 3 | 4682 | 35 | 0.7% | leiden | co-author |
| [Thomaes 2014 (180)](#_ENREF_180) | 171 | 5 | 749 | 15 | 2.0% | vu | co-author |
| [Tilbury 2014 (181)](#_ENREF_181) | 35 | 7 | 569 | 19 | 3.3% | leiden |  |
| [van Amerongen 2014 (182)](#_ENREF_182) | 38 | 6 | 5599 | 212 | 3.8% | nijmegen |  |
| [van Balkum 2016 (183)](#_ENREF_183) | 24 | 3 | 449 | 7 | 1.6% | utrecht |  |
| [van Beek 2014 (184)](#_ENREF_184) | 221 | 3 | 2224 | 21 | 0.9% | vu | co-author |
| [van Bokhorst-de van der Schueren 2014 (185)](#_ENREF_185) | 191 | 3 | 8313 | 24 | 0.3% | vu | co-author |
| [van Bokhorst-de van der Schueren 2014 (186)](#_ENREF_186) | 191 | 3 | 7357 | 83 | 1.1% | vu | co-author |
| [van Bunderen 2014 (187)](#_ENREF_187) | 16 | 4 | 168 | 2 | 1.2% | utrecht |  |
| [van Dalen 2016 (188)](#_ENREF_188) |  | 4 | 319 | 3 | 0.9% | amc | co-author |
| [van Dalen 2016 (189)](#_ENREF_189) |  | 3 | 4200 | 9 | 0.2% | amc |  |
| [van Dalen-Kok 2015 (190)](#_ENREF_190) | 90 | 3 | 1238 | 19 | 1.5% | nijmegen |  |
| [van de Glind 2014 (191)](#_ENREF_191) |  | 5 | 810 | 11 | 1.4% | amc |  |
| [van de Vorst 2016 (192)](#_ENREF_192) | 109 | 3 | 12703 | 12 | 0.1% | utrecht |  |
| [van den Berg 2014 (193)](#_ENREF_193) | 78 | 3 | 1596 | 1 | 0.1% | utrecht |  |
| [van den Broek 2014 (194)](#_ENREF_194) | 41 | 5 | 4647 | 33 | 0.7% | vu |  |
| [van den Dungen 2014 (195)](#_ENREF_195) | 43 | 4 | 741 | 14 | 1.9% | nijmegen |  |
| [van den Haak 2015 (196)](#_ENREF_196) | 9 | 8 | 742 | 29 | 3.9% | groningen |  |
| [van der Gijp 2016 (197)](#_ENREF_197) |  | 6 | 10555 | 22 | 0.2% | utrecht |  |
| [van der Have 2014 (198)](#_ENREF_198) | 45 | 5 | 2060 | 29 | 1.4% | utrecht |  |
| [van der Heijden 2014 (199)](#_ENREF_199) | 46 | 5 | 481 | 10 | 2.1% | amc |  |
| [van der Horst 2016 (200)](#_ENREF_200) | 20 | 4 | 1926 | 22 | 1.1% | vu |  |
| [van der Linden 2015 (201)](#_ENREF_201) | 82 | 5 | 1873 | 48 | 2.6% | leiden |  |
| [van der Meij 2016 (202)](#_ENREF_202) | 77 | 4 | 3779 | 33 | 0.9% | vu | co-author |
| [van der Pols-Vijlbrief 2014 (203)](#_ENREF_203) |  | 4 | 2481 | 19 | 0.8% | vu |  |
| [van der Steen 2014 (204)](#_ENREF_204) | 122 | 3 | 3899 | 3 | 0.1% | utrecht |  |
| [van der Voort 2015 (205)](#_ENREF_205) | 16 | 4 | 429 | 45 | 10.5% | leiden |  |
| [van Dongen 2016 (206)](#_ENREF_206) | 25 | 3 | 1197 | 40 | 3.3% | leiden |  |
| [van Drongelen 2014 (207)](#_ENREF_207) |  | 3 | 243 | 9 | 3.7% | vu |  |
| [van Duijn 2014 (208)](#_ENREF_208) | 18 | 1 |  | 49 |  | amc |  |
| [van Erp 2014 (209)](#_ENREF_209) |  | 8 | 459 | 4 | 0.9% | leiden |  |
| [van Galen 2015 (210)](#_ENREF_210) |  | 3 | 390 | 40 | 10.3% | amc |  |
| [van Haalen 2015 (211)](#_ENREF_211) | 60 | 2 |  |  |  | vu |  |
| [van Herwaarden 2015 (212)](#_ENREF_212) |  | 5 | 3825 | 16 | 0.4% | amc |  |
| [van Hulst 2016 (213)](#_ENREF_213) | 38 | 4 | 1533 | 47 | 3.1% | amc |  |
| [van Hulsteijn 2014 (214)](#_ENREF_214) |  | 8 | 1648 | 17 | 1.0% | leiden |  |
| [van Leeuwen 2015 (215)](#_ENREF_215) | 51 | 4 | 1298 | 28 | 2.2% | leiden |  |
| [van Litsenburg 2014 (216)](#_ENREF_216) | 122 | 5 | 2974 | 15 | 0.5% | vu | co-author |
| [van Loon 2014 (217)](#_ENREF_217) | 147 | 5 | 6605 | 23 | 0.3% | nijmegen |  |
| [van Meurs 2014 (218)](#_ENREF_218) | 17 | 4 | 662 | 12 | 1.8% | groningen |  |
| [van Nies 2014 (219)](#_ENREF_219) | 14 | 8 | 836 | 18 | 2.2% | leiden | co-author |
| [van Nieuwenhuizen 2015 (220)](#_ENREF_220) | 31 | 6 | 984 | 12 | 1.2% | amc | co-author |
| [van Oldenrijk 2014 (221)](#_ENREF_221) |  | 5 | 4299 | 44 | 1.0% | vu |  |
| [van Oostendorp 2016 (222)](#_ENREF_222) | 23 | 2 | 2692 | 12 | 0.4% | vu |  |
| [van Oostveen 2014 (223)](#_ENREF_223) | 100 | 6 | 5078 | 417 | 8.2% | leiden |  |
| [van Rijssen 2016 (224)](#_ENREF_224) |  | 3 | 3088 | 13 | 0.4% | amc |  |
| [van Tuyl 2014 (225)](#_ENREF_225) |  | 1 | 1941 | 351 | 18.1% | amc |  |
| [van Velzen 2014 (226)](#_ENREF_226) |  | 3 | 909 | 24 | 2.6% | amc | co-author |
| [van Vilsteren 2015 (227)](#_ENREF_227) | 157 | 3 | 5180 | 62 | 1.2% | leiden | co-author |
| [van Vlijmen 2016 (228)](#_ENREF_228) | 18 | 2 | 2027 | 15 | 0.7% | groningen |  |
| [van Wissen 2016 (229)](#_ENREF_229) |  | 2 | 2198 | 85 | 3.9% | nijmegen |  |
| [van Zuuren 2016 (230)](#_ENREF_230) |  | 10 |  | 47 |  | leiden | co-author |
| [Veerbeek 2014 (231)](#_ENREF_231) | 137 | 3 | 4173 | 1 | 0.0% | utrecht |  |
| [Vegting 2014 (232)](#_ENREF_232) |  | 5 | 1857 | 9 | 0.5% | vu | co-author |
| [Vehmeijer 2016 (233)](#_ENREF_233) | 211 | 3 | 1354 | 24 | 1.8% | amc | co-author |
| [Velzel 2015 (234)](#_ENREF_234) | 7 | 5 | 2872 | 12 | 0.4% | vu |  |
| [Verbeek-van Noord 2014 (235)](#_ENREF_235) | 50 | 3 | 637 | 7 | 1.1% | vu |  |
| [Verheggen 2016 (236)](#_ENREF_236) | 60 | 4 | 13168 | 117 | 0.9% | nijmegen |  |
| [Verlinden 2015 (237)](#_ENREF_237) | 37 | 3 | 260 | 12 | 4.6% | utrecht |  |
| [Verloop 2015 (238)](#_ENREF_238) | 57 | 2 | 675 | 10 | 1.5% | utrecht |  |
| [Verra 2015 (239)](#_ENREF_239) | 31 | 11 | 891 | 48 | 5.4% | amc |  |
| [Vissenberg 2015 (240)](#_ENREF_240) | 212 | 5 | 3290 | 19 | 0.6% | leiden |  |
| [Visser 2014 (241)](#_ENREF_241) | 60 | 2 | 1399 | 54 | 3.9% | utrecht |  |
| [Visser 2015 (242)](#_ENREF_242) |  | 3 | 3712 | 21 | 0.6% | amc |  |
| [Vlek 2016 (243)](#_ENREF_243) |  | 4 | 1971 | 9 | 0.5% | vu | co-author |
| [Vooijs 2015 (244)](#_ENREF_244) |  |  |  | 217 |  | leiden | co-author |
| [Vooijs 2015 (245)](#_ENREF_245) |  | 4 | 870 | 23 | 2.6% | amc |  |
| [Wang 2016 (246)](#_ENREF_246) | 43 | 5 | 1366 | 45 | 3.3% | vu | co-author |
| [Weber 2015 (247)](#_ENREF_247) | 52 | 15 | 1962 | 11 | 0.6% | leiden |  |
| [Wegner 2014 (248)](#_ENREF_248) |  | 3 | 657 | 17 | 2.6% | groningen |  |
| [Wegner 2014 (249)](#_ENREF_249) | 30 | 5 | 383 | 8 | 2.1% | utrecht |  |
| [Wegner 2015 (250)](#_ENREF_250) | 9 | 2 | 2418 | 38 | 1.6% | vu |  |
| [Wegner 2016 (251)](#_ENREF_251) | 187 | 6 | 1398 | 13 | 0.9% | leiden | co-author |
| [Wuister 2014 (252)](#_ENREF_252) | 42 | 2 |  | 65 |  | amc |  |
| [Yauw 2015 (253)](#_ENREF_253) | 30 | 4 | 3320 | 86 | 2.6% | groningen |  |
| [Zaal 2015 (254)](#_ENREF_254) |  | 4 | 11491 | 28 | 0.2% | vu |  |
| [Zaal-Schuller 2016 (255)](#_ENREF_255) | 50 | 6 |  |  |  | vu |  |
| [Zandbelt 2016 (256)](#_ENREF_256) | 387 | 2 | 1549 | 85 | 5.5% | leiden |  |
| [Zijlstra 2016 (257)](#_ENREF_257) | 27 | 2 | 1177 | 28 | 2.4% | groningen |  |
| [Zwinkels 2014 (258)](#_ENREF_258) | 34 | 2 | 3231 | 30 | 0.9% | amc |  |
|  |  |  |  |  |  |  |  |
| *Number of data points* | *189* | *256* | *246* | *253* | *241* | *258* | *45* |
| *Percentage of total* | *73%* | *99%* | *95%* | *98%* | *93%* | *100%* | *17%* |
|  |  |  |  |  |  |  |  |
| *minimum* | *6* | *1* | *74* | *0* | *0.02%* |  |  |
| *10 percentile* | *18* | *2* | *348* | *6* | *0.2%* |  |  |
| *25 percentile* | *29* | *3* | *674* | *11* | *0.7%* |  |  |
| *median* | *49* | *3* | *1534* | *20* | *1.4%* |  |  |
| *Average* | *78* | *3.8* | *2499* | *41* | *2.9%* |  |  |
| *75 percentile* | *90* | *5* | *3237* | *39* | *3.1%* |  |  |
| *90 percentile* | *189* | *6* | *5472* | *76* | *6.59%* |  |  |
| *maximum* | *558* | *20* | *17956* | *1342* | *59.5%* |  |  |

1. Abrahams HJ, Gielissen MF, Schmits IC, Verhagen CA, Rovers MM, Knoop H. Risk factors, prevalence, and course of severe fatigue after breast cancer treatment: a meta-analysis involving 12 327 breast cancer survivors. Annals of oncology : official journal of the European Society for Medical Oncology / ESMO. 2016 Jun;27(6):965-74.

2. Akintola AA, Jansen SW, van Bodegom D, van der Grond J, Westendorp RG, de Craen AJ, et al. Subclinical hypothyroidism and cognitive function in people over 60 years: a systematic review and meta-analysis. Frontiers in aging neuroscience. 2015;7:150.

3. Alam N, Hobbelink EL, van Tienhoven AJ, van de Ven PM, Jansma EP, Nanayakkara PW. The impact of the use of the Early Warning Score (EWS) on patient outcomes: a systematic review. Resuscitation. 2014 May;85(5):587-94.

4. Andela CD, Scharloo M, Pereira AM, Kaptein AA, Biermasz NR. Quality of life (QoL) impairments in patients with a pituitary adenoma: a systematic review of QoL studies. Pituitary. 2015 Oct;18(5):752-76.

5. Baltes TP, Zwiers R, Wiegerinck JI, van Dijk CN. Surgical treatment for midportion Achilles tendinopathy: a systematic review. Knee surgery, sports traumatology, arthroscopy : official journal of the ESSKA. 2016 Mar 12.

6. Batelaan NM, Seldenrijk A, Bot M, van Balkom AJ, Penninx BW. Anxiety and new onset of cardiovascular disease: critical review and meta-analysis. The British journal of psychiatry : the journal of mental science. 2016 Mar;208(3):223-31.

7. Beeldman E, Raaphorst J, Klein Twennaar M, de Visser M, Schmand BA, de Haan RJ. The cognitive profile of ALS: a systematic review and meta-analysis update. Journal of neurology, neurosurgery, and psychiatry. 2016 Jun;87(6):611-9.

8. Beishuizen CR, Stephan BC, van Gool WA, Brayne C, Peters RJ, Andrieu S, et al. Web-Based Interventions Targeting Cardiovascular Risk Factors in Middle-Aged and Older People: A Systematic Review and Meta-Analysis. Journal of medical Internet research. 2016;18(3):e55.

9. Bij de Vaate AJ, van der Voet LF, Naji O, Witmer M, Veersema S, Brolmann HA, et al. Prevalence, potential risk factors for development and symptoms related to the presence of uterine niches following Cesarean section: systematic review. Ultrasound in obstetrics & gynecology : the official journal of the International Society of Ultrasound in Obstetrics and Gynecology. 2014 Apr;43(4):372-82.

10. Bijker R, Agyemang C. The influence of early-life conditions on cardiovascular disease later in life among ethnic minority populations: a systematic review. Internal and emergency medicine. 2016 Apr;11(3):341-53.

11. Bittermann AJ, Wegner I, Noordman BJ, Vincent R, van der Heijden GJ, Grolman W. An introduction of genetics in otosclerosis: a systematic review. Otolaryngology--head and neck surgery : official journal of American Academy of Otolaryngology-Head and Neck Surgery. 2014 Jan;150(1):34-9.

12. Bleeker G, Tytgat GA, Adam JA, Caron HN, Kremer LC, Hooft L, et al. 123I-MIBG scintigraphy and 18F-FDG-PET imaging for diagnosing neuroblastoma. The Cochrane database of systematic reviews. 2015(9):CD009263.

13. Bleker SM, van Es N, van Gils L, Daams JG, Kleinjan A, Buller HR, et al. Clinical course of upper extremity deep vein thrombosis in patients with or without cancer: a systematic review. Thrombosis research. 2016 Apr;140 Suppl 1:S81-8.

14. Blikkendaal MD, Schepers EM, van Zwet EW, Twijnstra AR, Jansen FW. Hysterectomy in very obese and morbidly obese patients: a systematic review with cumulative analysis of comparative studies. Archives of gynecology and obstetrics. 2015 Oct;292(4):723-38.

15. Boender TS, Sigaloff KC, McMahon JH, Kiertiburanakul S, Jordan MR, Barcarolo J, et al. Long-term Virological Outcomes of First-Line Antiretroviral Therapy for HIV-1 in Low- and Middle-Income Countries: A Systematic Review and Meta-analysis. Clinical infectious diseases : an official publication of the Infectious Diseases Society of America. 2015 Nov 1;61(9):1453-61.

16. Boerebach BC, Scheepers RA, van der Leeuw RM, Heineman MJ, Arah OA, Lombarts KM. The impact of clinicians' personality and their interpersonal behaviors on the quality of patient care: a systematic review. International journal for quality in health care : journal of the International Society for Quality in Health Care / ISQua. 2014 Aug;26(4):426-81.

17. Boom V, Anton J, Lahdenne P, Quartier P, Ravelli A, Wulffraat NM, et al. Evidence-based diagnosis and treatment of macrophage activation syndrome in systemic juvenile idiopathic arthritis. Pediatr Rheumatol Online J. 2015;13:55.

18. Borgert MJ, Goossens A, Dongelmans DA. What are effective strategies for the implementation of care bundles on ICUs: a systematic review. Implementation science : IS. 2015;10:119.

19. Borsje P, Wetzels RB, Lucassen PL, Pot AM, Koopmans RT. The course of neuropsychiatric symptoms in community-dwelling patients with dementia: a systematic review. International psychogeriatrics / IPA. 2015 Mar;27(3):385-405.

20. Breugom AJ, Swets M, Bosset JF, Collette L, Sainato A, Cionini L, et al. Adjuvant chemotherapy after preoperative (chemo)radiotherapy and surgery for patients with rectal cancer: a systematic review and meta-analysis of individual patient data. The Lancet Oncology. 2015 Feb;16(2):200-7.

21. Broeder TP, Grooteman KV, Overdijkink SB, Selhorst CE, Kaper NM, Grolman W, et al. Inconclusive evidence that age predicts a prolonged or chronic course of acute rhinosinusitis in adults: a systematic review of the evidence base. Otolaryngology--head and neck surgery : official journal of American Academy of Otolaryngology-Head and Neck Surgery. 2014 Mar;150(3):365-70.

22. Broersen LH, Pereira AM, Jorgensen JO, Dekkers OM. Adrenal Insufficiency in Corticosteroids Use: Systematic Review and Meta-Analysis. The Journal of clinical endocrinology and metabolism. 2015 Jun;100(6):2171-80.

23. Brom L, Hopmans W, Pasman HR, Timmermans DR, Widdershoven GA, Onwuteaka-Philipsen BD. Congruence between patients' preferred and perceived participation in medical decision-making: a review of the literature. BMC medical informatics and decision making. 2014;14:25.

24. Brons S, van Beusichem ME, Bronkhorst EM, Draaisma JM, Berge SJ, Schols JG, et al. Methods to quantify soft tissue-based cranial growth and treatment outcomes in children: a systematic review. PLoS One. 2014;9(2):e89602.

25. Bruijnzeel H, Ziylan F, Stegeman I, Topsakal V, Grolman W. A Systematic Review to Define the Speech and Language Benefit of Early (<12 Months) Pediatric Cochlear Implantation. Audiology & neuro-otology. 2016;21(2):113-26.

26. Bruijnzeel H, Draaisma K, van Grootel R, Stegeman I, Topsakal V, Grolman W. Systematic Review on Surgical Outcomes and Hearing Preservation for Cochlear Implantation in Children and Adults. Otolaryngology--head and neck surgery : official journal of American Academy of Otolaryngology-Head and Neck Surgery. 2016 Apr;154(4):586-96.

27. Bruijnzeel H, van den Aardweg MT, Grolman W, Stegeman I, van der Veen EL. A systematic review on the surgical outcome of preauricular sinus excision techniques. The Laryngoscope. 2016 Jul;126(7):1535-44.

28. Brunsveld-Reinders AH, Arbous MS, De Vos R, De Jonge E. Incident and error reporting systems in intensive care: a systematic review of the literature. International journal for quality in health care : journal of the International Society for Quality in Health Care / ISQua. 2016 Feb;28(1):2-13.

29. Burger NB, Bekker MN, de Groot CJ, Christoffels VM, Haak MC. Why increased nuchal translucency is associated with congenital heart disease: a systematic review on genetic mechanisms. Prenatal diagnosis. 2015 Jun;35(6):517-28.

30. Busard C, Zweegers J, Limpens J, Langendam M, Spuls PI. Combined use of systemic agents for psoriasis: a systematic review. JAMA dermatology. 2014 Nov;150(11):1213-20.

31. Charehbili A, Fontein DB, Kroep JR, Liefers GJ, Mieog JS, Nortier JW, et al. Neoadjuvant hormonal therapy for endocrine sensitive breast cancer: a systematic review. Cancer treatment reviews. 2014 Feb;40(1):86-92.

32. Coelen RJ, Ruys AT, Besselink MG, Busch OR, van Gulik TM. Diagnostic accuracy of staging laparoscopy for detecting metastasized or locally advanced perihilar cholangiocarcinoma: a systematic review and meta-analysis. Surgical endoscopy. 2016 Feb 19.

33. Conijn AP, Jens S, Terwee CB, Breek JC, Koelemay MJ. Assessing the quality of available patient reported outcome measures for intermittent claudication: a systematic review using the COSMIN checklist. European journal of vascular and endovascular surgery : the official journal of the European Society for Vascular Surgery. 2015 Mar;49(3):316-34.

34. Danhof NA, Kamphuis EI, Limpens J, van Lonkhuijzen LR, Pajkrt E, Mol BW. The risk of preterm birth of treated versus untreated cervical intraepithelial neoplasia (CIN): a systematic review and meta-analysis. European journal of obstetrics, gynecology, and reproductive biology. 2015 May;188:24-33.

35. de Glas NA, Kiderlen M, de Craen AJ, Hamaker ME, Portielje JE, van de Velde CJ, et al. Assessing treatment effects in older breast cancer patients: systematic review of observational research methods. Cancer treatment reviews. 2015 Mar;41(3):254-61.

36. de Groof EJ, Sahami S, Lucas C, Ponsioen CY, Bemelman WA, Buskens CJ. Treatment of perianal fistula in Crohn's disease: a systematic review and meta-analysis comparing seton drainage and anti-tumour necrosis factor treatment. Colorectal disease : the official journal of the Association of Coloproctology of Great Britain and Ireland. 2016 Jul;18(7):667-75.

37. de Groof EJ, Cabral VN, Buskens CJ, Morton DG, Hahnloser D, Bemelman WA. Systematic review of evidence and consensus on perianal fistula: an analysis of national and international guidelines. Colorectal disease : the official journal of the Association of Coloproctology of Great Britain and Ireland. 2016 Apr;18(4):O119-34.

38. de Hundt M, Velzel J, de Groot CJ, Mol BW, Kok M. Mode of delivery after successful external cephalic version: a systematic review and meta-analysis. Obstetrics and gynecology. 2014 Jun;123(6):1327-34.

39. de Jong Y, Pinckaers JH, ten Brinck RM, Lycklama a Nijeholt AA, Dekkers OM. Urinating standing versus sitting: position is of influence in men with prostate enlargement. A systematic review and meta-analysis. PLoS One. 2014;9(7):e101320.

40. de Ruiter CM, van der Veer C, Leeflang MM, Deborggraeve S, Lucas C, Adams ER. Molecular tools for diagnosis of visceral leishmaniasis: systematic review and meta-analysis of diagnostic test accuracy. Journal of clinical microbiology. 2014 Sep;52(9):3147-55.

41. de Vries SG, Visser BJ, Nagel IM, Goris MG, Hartskeerl RA, Grobusch MP. Leptospirosis in Sub-Saharan Africa: a systematic review. International journal of infectious diseases : IJID : official publication of the International Society for Infectious Diseases. 2014 Nov;28:47-64.

42. de Vries RB, Leenaars M, Tra J, Huijbregtse R, Bongers E, Jansen JA, et al. The potential of tissue engineering for developing alternatives to animal experiments: a systematic review. J Tissue Eng Regen Med. 2015 Jul;9(7):771-8.

43. de Vries EE, van den Munckhof B, Braun KP, van Royen-Kerkhof A, de Jager W, Jansen FE. Inflammatory mediators in human epilepsy: A systematic review and meta-analysis. Neuroscience and biobehavioral reviews. 2016 Apr;63:177-90.

44. de Wit HM, Te Groen M, Rovers MM, Tack CJ. The placebo response of injectable GLP-1 receptor agonists vs. oral DPP-4 inhibitors and SGLT-2 inhibitors: a systematic review and meta-analysis. British journal of clinical pharmacology. 2016 Jul;82(1):301-14.

45. Delli K, Dijkstra PU, Stel AJ, Bootsma H, Vissink A, Spijkervet FK. Diagnostic properties of ultrasound of major salivary glands in Sjogren's syndrome: a meta-analysis. Oral diseases. 2015 Sep;21(6):792-800.

46. Demirdas S, Coakley KE, Bisschop PH, Hollak CE, Bosch AM, Singh RH. Bone health in phenylketonuria: a systematic review and meta-analysis. Orphanet journal of rare diseases. 2015;10:17.

47. Dietz SM, Tacke CE, Hutten BA, Kuijpers TW. Peripheral Endothelial (Dys)Function, Arterial Stiffness and Carotid Intima-Media Thickness in Patients after Kawasaki Disease: A Systematic Review and Meta-Analyses. PLoS One. 2015;10(7):e0130913.

48. Donker-Cools BH, Daams JG, Wind H, Frings-Dresen MH. Effective return-to-work interventions after acquired brain injury: A systematic review. Brain injury. 2016;30(2):113-31.

49. Driessen JP, van Kempen PM, van der Heijden GJ, Philippens ME, Pameijer FA, Stegeman I, et al. Diffusion-weighted imaging in head and neck squamous cell carcinomas: a systematic review. Head & neck. 2015 Mar;37(3):440-8.

50. Driessen SR, Sandberg EM, la Chapelle CF, Twijnstra AR, Rhemrev JP, Jansen FW. Case-Mix Variables and Predictors for Outcomes of Laparoscopic Hysterectomy: A Systematic Review. Journal of minimally invasive gynecology. 2016 Mar-Apr;23(3):317-30.

51. Dubois L, Steenen SA, Gooris PJ, Mourits MP, Becking AG. Controversies in orbital reconstruction--II. Timing of post-traumatic orbital reconstruction: a systematic review. International journal of oral and maxillofacial surgery. 2015 Apr;44(4):433-40.

52. Dubois L, Steenen SA, Gooris PJ, Mourits MP, Becking AG. Controversies in orbital reconstruction--I. Defect-driven orbital reconstruction: a systematic review. International journal of oral and maxillofacial surgery. 2015 Mar;44(3):308-15.

53. Ebisch RM, Rovers MM, Bosgraaf RP, van der Pluijm-Schouten HW, Melchers WJ, van den Akker PA, et al. Evidence supporting see-and-treat management of cervical intraepithelial neoplasia: a systematic review and meta-analysis. BJOG : an international journal of obstetrics and gynaecology. 2016 Jan;123(1):59-66.

54. Engelhardt EG, Garvelink MM, de Haes JH, van der Hoeven JJ, Smets EM, Pieterse AH, et al. Predicting and communicating the risk of recurrence and death in women with early-stage breast cancer: a systematic review of risk prediction models. Journal of clinical oncology : official journal of the American Society of Clinical Oncology. 2014 Jan 20;32(3):238-50.

55. Flik CE, van Rood YR, de Wit NJ. Systematic review: knowledge and educational needs of patients with irritable bowel syndrome. European journal of gastroenterology & hepatology. 2015 Apr;27(4):367-71.

56. Font-Gonzalez A, Mulder RL, Loeffen EA, Byrne J, van Dulmen-den Broeder E, van den Heuvel-Eibrink MM, et al. Fertility preservation in children, adolescents, and young adults with cancer: Quality of clinical practice guidelines and variations in recommendations. Cancer. 2016 Jul 15;122(14):2216-23.

57. Fransen J, Kazemi-Bajestani SM, Bredie SJ, Popa CD. Rheumatoid Arthritis Disadvantages Younger Patients for Cardiovascular Diseases: A Meta-Analysis. PLoS One. 2016;11(6):e0157360.

58. Frerichs KA, Nigten G, Romeijn K, Kaper NM, Grolman W, van der Heijden GJ. Inconclusive evidence for allergic rhinitis to predict a prolonged or chronic course of acute rhinosinusitis. Otolaryngology--head and neck surgery : official journal of American Academy of Otolaryngology-Head and Neck Surgery. 2014 Jan;150(1):22-7.

59. Garritsen FM, Brouwer MW, Limpens J, Spuls PI. Photo(chemo)therapy in the management of atopic dermatitis: an updated systematic review with implications for practice and research. Br J Dermatol. 2014 Mar;170(3):501-13.

60. Geboers B, Brainard JS, Loke YK, Jansen CJ, Salter C, Reijneveld SA, et al. The association of health literacy with adherence in older adults, and its role in interventions: a systematic meta-review. BMC public health. 2015;15:903.

61. Gerbens LA, Prinsen CA, Chalmers JR, Drucker AM, von Kobyletzki LB, Limpens J, et al. Evaluation of the measurement properties of symptom measurement instruments for atopic eczema: a systematic review. Allergy. 2016 Jun 20.

62. Gielen C, Dekkers O, Stijnen T, Schoones J, Brand A, Klautz R, et al. The effects of pre- and postoperative fibrinogen levels on blood loss after cardiac surgery: a systematic review and meta-analysis. Interactive cardiovascular and thoracic surgery. 2014 Mar;18(3):292-8.

63. Glaudemans JJ, Moll van Charante EP, Willems DL. Advance care planning in primary care, only for severely ill patients? A structured review. Family practice. 2015 Feb;32(1):16-26.

64. Gorter EA, Hamdy NA, Appelman-Dijkstra NM, Schipper IB. The role of vitamin D in human fracture healing: a systematic review of the literature. Bone. 2014 Jul;64:288-97.

65. Govaert JA, van Bommel AC, van Dijk WA, van Leersum NJ, Tollenaar RA, Wouters MW. Reducing healthcare costs facilitated by surgical auditing: a systematic review. World journal of surgery. 2015 Jul;39(7):1672-80.

66. Groenen KH, Pouw MH, Hannink G, Hosman AJ, van der Linden YM, Verdonschot N, et al. The effect of radiotherapy, and radiotherapy combined with bisphosphonates or RANK ligand inhibitors on bone quality in bone metastases. A systematic review. Radiotherapy and oncology : journal of the European Society for Therapeutic Radiology and Oncology. 2016 May;119(2):194-201.

67. Grooten IJ, Vinke ME, Roseboom TJ, Painter RC. A Systematic Review and Meta-Analysis of the Utility of Corticosteroids in the Treatment of Hyperemesis Gravidarum. Nutrition and metabolic insights. 2015;8(Suppl 1):23-32.

68. Gvozdenovic E, Koevoets R, Langenhoff J, Allaart C, Landewe RB. Comparison of characteristics of international and national databases for rheumatoid arthritis: a systematic literature review. Scandinavian journal of rheumatology. 2014;43(5):349-55.

69. Hamelinck VC, Bastiaannet E, Pieterse AH, Jannink I, van de Velde CJ, Liefers GJ, et al. Patients' preferences for surgical and adjuvant systemic treatment in early breast cancer: a systematic review. Cancer treatment reviews. 2014 Sep;40(8):1005-18.

70. Harmsen AM, Giannakopoulos GF, Moerbeek PR, Jansma EP, Bonjer HJ, Bloemers FW. The influence of prehospital time on trauma patients outcome: a systematic review. Injury. 2015 Apr;46(4):602-9.

71. Harmsen RT, Haanstra TM, Sierevelt IN, Jansma EP, Nolte PA, Nicolai MP, et al. Does total hip replacement affect sexual quality of life? BMC musculoskeletal disorders. 2016;17(1):198.

72. Haroun D, Smits F, van Etten-Jamaludin F, Schene A, van Weert H, Ter Riet G. The effects of interventions on quality of life, morbidity and consultation frequency in frequent attenders in primary care: A systematic review. The European journal of general practice. 2016 Jun;22(2):71-82.

73. Hassell ME, Hildick-Smith D, Durand E, Kikkert WJ, Wiegerinck EM, Stabile E, et al. Antiplatelet therapy following transcatheter aortic valve implantation. Heart (British Cardiac Society). 2015 Jul;101(14):1118-25.

74. Hauer AJ, Luiten EL, van Erp NF, Blase PE, Aarts MC, Kaper NM, et al. No evidence for distinguishing bacterial from viral acute rhinosinusitis using fever and facial/dental pain: a systematic review of the evidence base. Otolaryngology--head and neck surgery : official journal of American Academy of Otolaryngology-Head and Neck Surgery. 2014 Jan;150(1):28-33.

75. Havermans BM, Schlevis RM, Boot CR, Brouwers EP, Anema JR, van der Beek AJ. Process variables in organizational stress management intervention evaluation research: a systematic review. Scandinavian journal of work, environment & health. 2016 May 11.

76. Heida KY, Velthuis BK, Oudijk MA, Reitsma JB, Bots ML, Franx A, et al. Cardiovascular disease risk in women with a history of spontaneous preterm delivery: A systematic review and meta-analysis. European journal of preventive cardiology. 2016 Feb;23(3):253-63.

77. Heitink-Polle KM, Nijsten J, Boonacker CW, de Haas M, Bruin MC. Clinical and laboratory predictors of chronic immune thrombocytopenia in children: a systematic review and meta-analysis. Blood. 2014 Nov 20;124(22):3295-307.

78. Helmerhorst HJ, Roos-Blom MJ, van Westerloo DJ, de Jonge E. Association Between Arterial Hyperoxia and Outcome in Subsets of Critical Illness: A Systematic Review, Meta-Analysis, and Meta-Regression of Cohort Studies. Critical care medicine. 2015 Jul;43(7):1508-19.

79. Hentschel MA, Huizinga P, van der Velden DL, Wegner I, Bittermann AJ, van der Heijden GJ, et al. Limited evidence for the effect of sodium fluoride on deterioration of hearing loss in patients with otosclerosis: a systematic review of the literature. Otology & neurotology : official publication of the American Otological Society, American Neurotology Society [and] European Academy of Otology and Neurotology. 2014 Jul;35(6):1052-7.

80. Hoencamp R, Vermetten E, Tan EC, Putter H, Leenen LP, Hamming JF. Systematic review of the prevalence and characteristics of battle casualties from NATO coalition forces in Iraq and Afghanistan. Injury. 2014 Jul;45(7):1028-34.

81. Hofstede SN, Nouta KA, Jacobs W, van Hooff ML, Wymenga AB, Pijls BG, et al. Mobile bearing vs fixed bearing prostheses for posterior cruciate retaining total knee arthroplasty for postoperative functional status in patients with osteoarthritis and rheumatoid arthritis. The Cochrane database of systematic reviews. 2015(2):CD003130.

82. Hofstede SN, Gademan MG, Vliet Vlieland TP, Nelissen RG, Marang-van de Mheen PJ. Preoperative predictors for outcomes after total hip replacement in patients with osteoarthritis: a systematic review. BMC musculoskeletal disorders. 2016;17(1):212.

83. Hoogedoorn L, Peppelman M, van de Kerkhof PC, van Erp PE, Gerritsen MJ. The value of in vivo reflectance confocal microscopy in the diagnosis and monitoring of inflammatory and infectious skin diseases: a systematic review. Br J Dermatol. 2015;172(5):1222-48.

84. Hooker A, Fraenk D, Brolmann H, Huirne J. Prevalence of intrauterine adhesions after termination of pregnancy: a systematic review. The European journal of contraception & reproductive health care : the official journal of the European Society of Contraception. 2016 Aug;21(4):329-35.

85. Horbach SE, Rigter IM, Smitt JH, Reekers JA, Spuls PI, van der Horst CM. Intralesional Bleomycin Injections for Vascular Malformations: A Systematic Review and Meta-Analysis. Plastic and reconstructive surgery. 2016 Jan;137(1):244-56.

86. Huisman MG, Kok M, de Bock GH, van Leeuwen BL. Delivering tailored surgery to older cancer patients: Preoperative geriatric assessment domains and screening tools - A systematic review of systematic reviews. European journal of surgical oncology : the journal of the European Society of Surgical Oncology and the British Association of Surgical Oncology. 2016 Jun 21.

87. Ingelse SA, Wosten-van Asperen RM, Lemson J, Daams JG, Bem RA, van Woensel JB. Pediatric Acute Respiratory Distress Syndrome: Fluid Management in the PICU. Frontiers in pediatrics. 2016;4:21.

88. Inklaar MJ, van Klink JM, Stolk TT, van Zwet EW, Oepkes D, Lopriore E. Cerebral injury in monochorionic twins with selective intrauterine growth restriction: a systematic review. Prenatal diagnosis. 2014 Mar;34(3):205-13.

89. Jalalzadeh H, Indrakusuma R, Planken RN, Legemate DA, Koelemay MJ, Balm R. Inflammation as a Predictor of Abdominal Aortic Aneurysm Growth and Rupture: A Systematic Review of Imaging Biomarkers. European journal of vascular and endovascular surgery : the official journal of the European Society for Vascular Surgery. 2016 Jun 6.

90. Jansen FA, Blumenfeld YJ, Fisher A, Cobben JM, Odibo AO, Borrell A, et al. Array comparative genomic hybridization and fetal congenital heart defects: a systematic review and meta-analysis. Ultrasound in obstetrics & gynecology : the official journal of the International Society of Ultrasound in Obstetrics and Gynecology. 2015 Jan;45(1):27-35.

91. Jansen S, Bhangu J, de Rooij S, Daams J, Kenny RA, van der Velde N. The Association of Cardiovascular Disorders and Falls: A Systematic Review. Journal of the American Medical Directors Association. 2016 Mar 1;17(3):193-9.

92. Jens S, Conijn AP, Koelemay MJ, Bipat S, Reekers JA. Randomized trials for endovascular treatment of infrainguinal arterial disease: systematic review and meta-analysis (Part 1: Above the knee). European journal of vascular and endovascular surgery : the official journal of the European Society for Vascular Surgery. 2014 May;47(5):524-35.

93. Jilesen AP, van Eijck CH, in't Hof KH, van Dieren S, Gouma DJ, van Dijkum EJ. Postoperative Complications, In-Hospital Mortality and 5-Year Survival After Surgical Resection for Patients with a Pancreatic Neuroendocrine Tumor: A Systematic Review. World journal of surgery. 2016 Mar;40(3):729-48.

94. Jonker SJ, Menting TP, Warle MC, Ritskes-Hoitinga M, Wever KE. Preclinical Evidence for the Efficacy of Ischemic Postconditioning against Renal Ischemia-Reperfusion Injury, a Systematic Review and Meta-Analysis. PLoS One. 2016;11(3):e0150863.

95. Kabaghe AN, Visser BJ, Spijker R, Phiri KS, Grobusch MP, van Vugt M. Health workers' compliance to rapid diagnostic tests (RDTs) to guide malaria treatment: a systematic review and meta-analysis. Malaria journal. 2016;15:163.

96. Kadouch DJ, Schram ME, Leeflang MM, Limpens J, Spuls PI, de Rie MA. In vivo confocal microscopy of basal cell carcinoma: a systematic review of diagnostic accuracy. Journal of the European Academy of Dermatology and Venereology : JEADV. 2015 Oct;29(10):1890-7.

97. Kamalski DM, Wegner I, Tange RA, Vincent R, Stegeman I, van der Heijden GJ, et al. Outcomes of different laser types in laser-assisted stapedotomy: a systematic review. Otology & neurotology : official publication of the American Otological Society, American Neurotology Society [and] European Academy of Otology and Neurotology. 2014 Jul;35(6):1046-51.

98. Kampshoff CS, Jansen F, van Mechelen W, May AM, Brug J, Chinapaw MJ, et al. Determinants of exercise adherence and maintenance among cancer survivors: a systematic review. The international journal of behavioral nutrition and physical activity. 2014;11:80.

99. Kaptein AA, Schoones JW, Fischer MJ, Thong MS, Kroep JR, van der Hoeven KJ. Illness Perceptions in Women with Breast Cancer-a Systematic Literature Review. Current breast cancer reports. 2015;7(3):117-26.

100. Kazemier BM, Buijs PE, Mignini L, Limpens J, de Groot CJ, Mol BW. Impact of obstetric history on the risk of spontaneous preterm birth in singleton and multiple pregnancies: a systematic review. BJOG : an international journal of obstetrics and gynaecology. 2014 Sep;121(10):1197-208; discussion 209.

101. Kernkamp WA, Verra WC, Pijls BG, Schoones JW, van der Linden HM, Nelissen RG. Conversion from knee arthrodesis to arthroplasty: systematic review. International orthopaedics. 2016 Mar 16.

102. Keurentjes JC, Pijls BG, Van Tol FR, Mentink JF, Mes SD, Schoones JW, et al. Which implant should we use for primary total hip replacement? A systematic review and meta-analysis. The Journal of bone and joint surgery American volume. 2014 Dec 17;96 Suppl 1:79-97.

103. Khorsand N, Kooistra HA, van Hest RM, Veeger NJ, Meijer K. A systematic review of prothrombin complex concentrate dosing strategies to reverse vitamin K antagonist therapy. Thrombosis research. 2015 Jan;135(1):9-19.

104. Klaassen KM, Dulak MG, van de Kerkhof PC, Pasch MC. The prevalence of onychomycosis in psoriatic patients: a systematic review. Journal of the European Academy of Dermatology and Venereology : JEADV. 2014 May;28(5):533-41.

105. Klein Hesselink EN, Steenvoorden D, Kapiteijn E, Corssmit EP, van der Horst-Schrivers AN, Lefrandt JD, et al. Therapy of endocrine disease: response and toxicity of small-molecule tyrosine kinase inhibitors in patients with thyroid carcinoma: a systematic review and meta-analysis. European journal of endocrinology / European Federation of Endocrine Societies. 2015 May;172(5):R215-25.

106. Kluitenberg B, van Middelkoop M, Diercks R, van der Worp H. What are the Differences in Injury Proportions Between Different Populations of Runners? A Systematic Review and Meta-Analysis. Sports medicine (Auckland, NZ). 2015 Aug;45(8):1143-61.

107. Kodde IF, Baerveldt RC, Mulder PG, Eygendaal D, van den Bekerom MP. Refixation techniques and approaches for distal biceps tendon ruptures: a systematic review of clinical studies. Journal of shoulder and elbow surgery / American Shoulder and Elbow Surgeons [et al]. 2016 Feb;25(2):e29-37.

108. Korevaar DA, Bossuyt PM, Hooft L. Infrequent and incomplete registration of test accuracy studies: analysis of recent study reports. BMJ open. 2014;4(1):e004596.

109. Korevaar DA, van Enst WA, Spijker R, Bossuyt PM, Hooft L. Reporting quality of diagnostic accuracy studies: a systematic review and meta-analysis of investigations on adherence to STARD. Evidence-based medicine. 2014 Apr;19(2):47-54.

110. Korterink JJ, Diederen K, Benninga MA, Tabbers MM. Epidemiology of pediatric functional abdominal pain disorders: a meta-analysis. PLoS One. 2015;10(5):e0126982.

111. Kroon FP, Rubio R, Schoones JW, Kloppenburg M. Intra-Articular Therapies in the Treatment of Hand Osteoarthritis: A Systematic Literature Review. Drugs & aging. 2016 Feb;33(2):119-33.

112. Kuijpers MA, Chiu YT, Nada RM, Carels CE, Fudalej PS. Three-dimensional imaging methods for quantitative analysis of facial soft tissues and skeletal morphology in patients with orofacial clefts: a systematic review. PLoS One. 2014;9(4):e93442.

113. Kuizenga-Wessel S, Benninga MA, Tabbers MM. Reporting outcome measures of functional constipation in children from 0 to 4 years of age. Journal of pediatric gastroenterology and nutrition. 2015 Apr;60(4):446-56.

114. Kuizenga-Wessel S, Heckert SL, Tros W, van Etten-Jamaludin FS, Benninga MA, Tabbers MM. Reporting on Outcome Measures of Functional Constipation in Children-A Systematic Review. Journal of pediatric gastroenterology and nutrition. 2016 Jun;62(6):840-6.

115. la Chapelle CF, Veersema S, Brolmann HA, Jansen FW. Effectiveness and feasibility of hysteroscopic sterilization techniques: a systematic review and meta-analysis. Fertility and sterility. 2015 Jun;103(6):1516-25 e1-3.

116. Labots M, Schutte LM, van der Mijn JC, Pham TV, Jimenez CR, Verheul HM. Mass spectrometry-based serum and plasma peptidome profiling for prediction of treatment outcome in patients with solid malignancies. The oncologist. 2014 Oct;19(10):1028-39.

117. Lambers Heerspink FO, Dorrestijn O, van Raay JJ, Diercks RL. Specific patient-related prognostic factors for rotator cuff repair: a systematic review. Journal of shoulder and elbow surgery / American Shoulder and Elbow Surgeons [et al]. 2014 Jul;23(7):1073-80.

118. Lantinga MA, Geudens A, Gevers TJ, Drenth JP. Systematic review: the management of hepatic cyst infection. Alimentary pharmacology & therapeutics. 2015 Feb;41(3):253-61.

119. Lemmers M, Verschoor MA, Hooker AB, Opmeer BC, Limpens J, Huirne JA, et al. Dilatation and curettage increases the risk of subsequent preterm birth: a systematic review and meta-analysis. Human reproduction (Oxford, England). 2016 Jan;31(1):34-45.

120. Lo-Fo-Wong DN, Sitnikova K, Sprangers MA, de Haes HC. Predictors of Health Care Use of Women with Breast Cancer: A Systematic Review. The breast journal. 2015 Sep-Oct;21(5):508-13.

121. Mallee WH, Henny EP, van Dijk CN, Kamminga SP, van Enst WA, Kloen P. Clinical diagnostic evaluation for scaphoid fractures: a systematic review and meta-analysis. The Journal of hand surgery. 2014 Sep;39(9):1683-91 e2.

122. Mallee WH, Wang J, Poolman RW, Kloen P, Maas M, de Vet HC, et al. Computed tomography versus magnetic resonance imaging versus bone scintigraphy for clinically suspected scaphoid fractures in patients with negative plain radiographs. The Cochrane database of systematic reviews. 2015(6):CD010023.

123. Man PW, van der Meer IM, Lips P, Middelkoop BJ. Vitamin D status and bone mineral density in the Chinese population: a review. Archives of osteoporosis. 2016;11:14.

124. Meiboom AA, de Vries H, Hertogh CM, Scheele F. Why medical students do not choose a career in geriatrics: a systematic review. BMC medical education. 2015;15:101.

125. Meijer MF, Reininga IH, Boerboom AL, Bulstra SK, Stevens M. Does imageless computer-assisted TKA lead to improved rotational alignment or fewer outliers? A systematic review. Clinical orthopaedics and related research. 2014 Oct;472(10):3124-33.

126. Menting SP, Dekker PM, Limpens J, Hooft L, Spuls PI. Methotrexate Dosing Regimen for Plaque-type Psoriasis: A Systematic Review of the Use of Test-dose, Start-dose, Dosing Scheme, Dose Adjustments, Maximum Dose and Folic Acid Supplementation. Acta dermato-venereologica. 2016 Jan;96(1):23-8.

127. Metsaars WP, Nagels J, Pijls BG, Langenhoff JM, Nelissen RG. Treatment of supination deformity for obstetric brachial plexus injury: a systematic review and meta-analysis. The Journal of hand surgery. 2014 Oct;39(10):1948-58 e2.

128. Meuleman T, Lashley LE, Dekkers OM, van Lith JM, Claas FH, Bloemenkamp KW. HLA associations and HLA sharing in recurrent miscarriage: A systematic review and meta-analysis. Human immunology. 2015 May;76(5):362-73.

129. Mulder FE, Hakvoort RA, Schoffelmeer MA, Limpens J, Van der Post JA, Roovers JP. Postpartum urinary retention: a systematic review of adverse effects and management. International urogynecology journal. 2014 Dec;25(12):1605-12.

130. Mulder RL, Paulides M, Langer T, Kremer LC, van Dalen EC. Cyclophosphamide versus ifosfamide for paediatric and young adult bone and soft tissue sarcoma patients. The Cochrane database of systematic reviews. 2015(9):CD006300.

131. Muntingh AD, van der Feltz-Cornelis CM, van Marwijk HW, Spinhoven P, van Balkom AJ. Collaborative care for anxiety disorders in primary care: a systematic review and meta-analysis. BMC family practice. 2016;17(1):62.

132. Nauta J, van Mechelen W, Otten RH, Verhagen EA. A systematic review on the effectiveness of school and community-based injury prevention programmes on risk behaviour and injury risk in 8-12 year old children. Journal of science and medicine in sport / Sports Medicine Australia. 2014 Mar;17(2):165-72.

133. Navarro-Compan V, Gherghe AM, Smolen JS, Aletaha D, Landewe R, van der Heijde D. Relationship between disease activity indices and their individual components and radiographic progression in RA: a systematic literature review. Rheumatology (Oxford, England). 2015 Jun;54(6):994-1007.

134. Negenborn VL, Groen JW, Smit JM, Niessen FB, Mullender MG. The Use of Autologous Fat Grafting for Treatment of Scar Tissue and Scar-Related Conditions: A Systematic Review. Plastic and reconstructive surgery. 2016 Jan;137(1):31e-43e.

135. Niemeijer ND, Alblas G, van Hulsteijn LT, Dekkers OM, Corssmit EP. Chemotherapy with cyclophosphamide, vincristine and dacarbazine for malignant paraganglioma and pheochromocytoma: systematic review and meta-analysis. Clinical endocrinology. 2014 Nov;81(5):642-51.

136. Ochodo EA, van Enst WA, Naaktgeboren CA, de Groot JA, Hooft L, Moons KG, et al. Incorporating quality assessments of primary studies in the conclusions of diagnostic accuracy reviews: a cross-sectional study. BMC medical research methodology. 2014;14:33.

137. Ochodo EA, Gopalakrishna G, Spek B, Reitsma JB, van Lieshout L, Polman K, et al. Circulating antigen tests and urine reagent strips for diagnosis of active schistosomiasis in endemic areas. The Cochrane database of systematic reviews. 2015(3):CD009579.

138. Olde Engberink RH, Frenkel WJ, van den Bogaard B, Brewster LM, Vogt L, van den Born BJ. Effects of thiazide-type and thiazide-like diuretics on cardiovascular events and mortality: systematic review and meta-analysis. Hypertension (Dallas, Tex : 1979). 2015 May;65(5):1033-40.

139. Olthof M, Stevens M, Bulstra SK, van den Akker-Scheek I. The association between comorbidity and length of hospital stay and costs in total hip arthroplasty patients: a systematic review. The Journal of arthroplasty. 2014 May;29(5):1009-14.

140. Onrust SA, Otten R, Lammers J, Smit F. School-based programmes to reduce and prevent substance use in different age groups: What works for whom? Systematic review and meta-regression analysis. Clinical psychology review. 2016 Mar;44:45-59.

141. Overdevest G, Vleggeert-Lankamp C, Jacobs W, Thome C, Gunzburg R, Peul W. Effectiveness of posterior decompression techniques compared with conventional laminectomy for lumbar stenosis. European spine journal : official publication of the European Spine Society, the European Spinal Deformity Society, and the European Section of the Cervical Spine Research Society. 2015 Oct;24(10):2244-63.

142. Owusu ED, Visser BJ, Nagel IM, Mens PF, Grobusch MP. The interaction between sickle cell disease and HIV infection: a systematic review. Clinical infectious diseases : an official publication of the Infectious Diseases Society of America. 2015 Feb 15;60(4):612-26.

143. Paap D, Takken T. Reference values for cardiopulmonary exercise testing in healthy adults: a systematic review. Expert review of cardiovascular therapy. 2014 Dec;12(12):1439-53.

144. Peeters SH, Devlieger R, Middeldorp JM, DeKoninck P, Deprest J, Lopriore E, et al. Fetal surgery in complicated monoamniotic pregnancies: case series and systematic review of the literature. Prenatal diagnosis. 2014 Jun;34(6):586-91.

145. Peters BJ, Janssen VE, Schramel FM, van de Garde EM. Quantitative and qualitative assessment of real world data comparative effectiveness research of systemic therapies in lung oncology: A systematic review. Cancer epidemiology. 2016 Jul 21;44:5-15.

146. Pijls BG, Meessen JM, Schoones JW, Fiocco M, van der Heide HJ, Sedrakyan A, et al. Increased Mortality in Metal-on-Metal versus Non-Metal-on-Metal Primary Total Hip Arthroplasty at 10 Years and Longer Follow-Up: A Systematic Review and Meta-Analysis. PLoS One. 2016;11(6):e0156051.

147. Proper KI, van de Langenberg D, Rodenburg W, Vermeulen RC, van der Beek AJ, van Steeg H, et al. The Relationship Between Shift Work and Metabolic Risk Factors: A Systematic Review of Longitudinal Studies. American journal of preventive medicine. 2016 May;50(5):e147-57.

148. Prosman GJ, Lo Fo Wong SH, van der Wouden JC, Lagro-Janssen AL. Effectiveness of home visiting in reducing partner violence for families experiencing abuse: a systematic review. Family practice. 2015 Jun;32(3):247-56.

149. Rashaan ZM, Krijnen P, Klamer RR, Schipper IB, Dekkers OM, Breederveld RS. Nonsilver treatment vs. silver sulfadiazine in treatment of partial-thickness burn wounds in children: a systematic review and meta-analysis. Wound repair and regeneration : official publication of the Wound Healing Society [and] the European Tissue Repair Society. 2014 Jul-Aug;22(4):473-82.

150. Rashid AN, Taminiau JA, Benninga MA, Saps M, Tabbers MM. Definitions and Outcome Measures in Pediatric Functional Upper Gastrointestinal Tract Disorders: A Systematic Review. Journal of pediatric gastroenterology and nutrition. 2016 Apr;62(4):581-7.

151. Reinink H, Wegner I, Stegeman I, Grolman W. Rapid systematic review of repeated application of the epley maneuver for treating posterior BPPV. Otolaryngology--head and neck surgery : official journal of American Academy of Otolaryngology-Head and Neck Surgery. 2014 Sep;151(3):399-406.

152. Roach RE, Helmerhorst FM, Lijfering WM, Stijnen T, Algra A, Dekkers OM. Combined oral contraceptives: the risk of myocardial infarction and ischemic stroke. The Cochrane database of systematic reviews. 2015(8):CD011054.

153. Roekevisch E, Spuls PI, Kuester D, Limpens J, Schmitt J. Efficacy and safety of systemic treatments for moderate-to-severe atopic dermatitis: a systematic review. The Journal of allergy and clinical immunology. 2014 Feb;133(2):429-38.

154. Rostamian S, Mahinrad S, Stijnen T, Sabayan B, de Craen AJ. Cognitive impairment and risk of stroke: a systematic review and meta-analysis of prospective cohort studies. Stroke; a journal of cerebral circulation. 2014 May;45(5):1342-8.

155. Ruiter L, Kok N, Limpens J, Derks JB, de Graaf IM, Mol BW, et al. Systematic review of accuracy of ultrasound in the diagnosis of vasa previa. Ultrasound in obstetrics & gynecology : the official journal of the International Society of Ultrasound in Obstetrics and Gynecology. 2015 May;45(5):516-22.

156. Ruiter L, Kok N, Limpens J, Derks JB, de Graaf IM, Mol B, et al. Incidence of and risk indicators for vasa praevia: a systematic review. BJOG : an international journal of obstetrics and gynaecology. 2016 Jul;123(8):1278-87.

157. Ruys AT, Groot Koerkamp B, Wiggers JK, Klumpen HJ, ten Kate FJ, van Gulik TM. Prognostic biomarkers in patients with resected cholangiocarcinoma: a systematic review and meta-analysis. Annals of surgical oncology. 2014 Feb;21(2):487-500.

158. San Giorgi MR, Helder HM, Lindeman RS, de Bock GH, Dikkers FG. The association between gastroesophageal reflux disease and recurrent respiratory papillomatosis: A systematic review. The Laryngoscope. 2016 Apr 26.

159. Sarac C, Duijnisveld BJ, van der Weide A, Schoones JW, Malessy MJ, Nelissen RG, et al. Outcome measures used in clinical studies on neonatal brachial plexus palsy: A systematic literature review using the International Classification of Functioning, Disability and Health. Journal of pediatric rehabilitation medicine. 2015;8(3):167-85; quiz 85-6.

160. Schaafsma FG, Mahmud N, Reneman MF, Fassier JB, Jungbauer FH. Pre-employment examinations for preventing injury, disease and sick leave in workers. The Cochrane database of systematic reviews. 2016(1):CD008881.

161. Scheper MC, Juul-Kristensen B, Rombaut L, Rameckers EA, Verbunt J, Engelbert RH. Disability in Adolescents and Adults Diagnosed With Hypermobility-Related Disorders: A Meta-Analysis. Archives of physical medicine and rehabilitation. 2016 Mar 11.

162. Schimmer JA, van der Steeg AF, Zuidema WP. Splenic function after angioembolization for splenic trauma in children and adults: A systematic review. Injury. 2016 Mar;47(3):525-30.

163. Schipper K, Bakker M, De Wit M, Ket JC, Abma TA. Strategies for disseminating recommendations or guidelines to patients: a systematic review. Implementation science : IS. 2016;11(1):82.

164. Schopman JE, Simon AC, Hoefnagel SJ, Hoekstra JB, Scholten RJ, Holleman F. The incidence of mild and severe hypoglycaemia in patients with type 2 diabetes mellitus treated with sulfonylureas: a systematic review and meta-analysis. Diabetes/metabolism research and reviews. 2014 Jan;30(1):11-22.

165. Schrijver EJ, de Graaf K, de Vries OJ, Maier AB, Nanayakkara PW. Efficacy and safety of haloperidol for in-hospital delirium prevention and treatment: A systematic review of current evidence. European journal of internal medicine. 2016 Jan;27:14-23.

166. Schuts EC, Hulscher ME, Mouton JW, Verduin CM, Stuart JW, Overdiek HW, et al. Current evidence on hospital antimicrobial stewardship objectives: a systematic review and meta-analysis. The Lancet Infectious diseases. 2016 Jul;16(7):847-56.

167. Sloothaak DA, Sahami S, van der Zaag-Loonen HJ, van der Zaag ES, Tanis PJ, Bemelman WA, et al. The prognostic value of micrometastases and isolated tumour cells in histologically negative lymph nodes of patients with colorectal cancer: a systematic review and meta-analysis. European journal of surgical oncology : the journal of the European Society of Surgical Oncology and the British Association of Surgical Oncology. 2014 Mar;40(3):263-9.

168. Smeets XJ, da Costa DW, Besselink MG, Bruno MJ, Fockens P, Mulder CJ, et al. Systematic review: periprocedural hydration in the prevention of post-ERCP pancreatitis. Alimentary pharmacology & therapeutics. 2016 Jul 22.

169. Smeulers M, Verweij L, Maaskant JM, de Boer M, Krediet CT, Nieveen van Dijkum EJ, et al. Quality indicators for safe medication preparation and administration: a systematic review. PLoS One. 2015;10(4):e0122695.

170. Smits MJ, van Wijk MP, Langendam MW, Benninga MA, Tabbers MM. Association between gastroesophageal reflux and pathologic apneas in infants: a systematic review. Neurogastroenterology and motility : the official journal of the European Gastrointestinal Motility Society. 2014 Nov;26(11):1527-38.

171. Steenen SA, van Wijk AJ, van der Heijden GJ, van Westrhenen R, de Lange J, de Jongh A. Propranolol for the treatment of anxiety disorders: Systematic review and meta-analysis. Journal of psychopharmacology (Oxford, England). 2016 Feb;30(2):128-39.

172. Steutel NF, Benninga MA, Langendam MW, de Kruijff I, Tabbers MM. Reporting outcome measures in trials of infant colic. Journal of pediatric gastroenterology and nutrition. 2014 Sep;59(3):341-6.

173. Stoekenbroek RM, Santema TB, Legemate DA, Ubbink DT, van den Brink A, Koelemay MJ. Hyperbaric oxygen for the treatment of diabetic foot ulcers: a systematic review. European journal of vascular and endovascular surgery : the official journal of the European Society for Vascular Surgery. 2014 Jun;47(6):647-55.

174. Storm-Versloot MN, Verweij L, Lucas C, Ludikhuize J, Goslings JC, Legemate DA, et al. Clinical relevance of routinely measured vital signs in hospitalized patients: a systematic review. Journal of nursing scholarship : an official publication of Sigma Theta Tau International Honor Society of Nursing / Sigma Theta Tau. 2014 Jan;46(1):39-49.

175. Straatman J, Harmsen AM, Cuesta MA, Berkhof J, Jansma EP, van der Peet DL. Predictive Value of C-Reactive Protein for Major Complications after Major Abdominal Surgery: A Systematic Review and Pooled-Analysis. PLoS One. 2015;10(7):e0132995.

176. Sutterland AL, Fond G, Kuin A, Koeter MW, Lutter R, van Gool T, et al. Beyond the association. Toxoplasma gondii in schizophrenia, bipolar disorder, and addiction: systematic review and meta-analysis. Acta psychiatrica Scandinavica. 2015 Sep;132(3):161-79.

177. Teepen JC, de Vroom SL, van Leeuwen FE, Tissing WJ, Kremer LC, Ronckers CM. Risk of subsequent gastrointestinal cancer among childhood cancer survivors: A systematic review. Cancer treatment reviews. 2016 Feb;43:92-103.

178. Terpstra ML, Aman J, van Nieuw Amerongen GP, Groeneveld AB. Plasma biomarkers for acute respiratory distress syndrome: a systematic review and meta-analysis*. Critical care medicine. 2014 Mar;42(3):691-700.

179. Theunissen SC, Rieffe C, Netten AP, Briaire JJ, Soede W, Schoones JW, et al. Psychopathology and its risk and protective factors in hearing-impaired children and adolescents: a systematic review. JAMA pediatrics. 2014 Feb;168(2):170-7.

180. Thomaes K, Dorrepaal E, Draijer N, Jansma EP, Veltman DJ, van Balkom AJ. Can pharmacological and psychological treatment change brain structure and function in PTSD? A systematic review. Journal of psychiatric research. 2014 Mar;50:1-15.

181. Tilbury C, Schaasberg W, Plevier JW, Fiocco M, Nelissen RG, Vliet Vlieland TP. Return to work after total hip and knee arthroplasty: a systematic review. Rheumatology (Oxford, England). 2014 Mar;53(3):512-25.

182. van Amerongen MJ, Boogaarts HD, de Vries J, Verbeek AL, Meijer FJ, Prokop M, et al. MRA versus DSA for follow-up of coiled intracranial aneurysms: a meta-analysis. AJNR Am J Neuroradiol. 2014 Sep;35(9):1655-61.

183. van Balkum M, Buijs B, Donselaar EJ, Erkelens DC, Goulin Lippi Fernandes E, Wegner I, et al. Systematic review of the diagnostic value of laryngeal stroboscopy in excluding early glottic carcinoma. Clinical otolaryngology : official journal of ENT-UK ; official journal of Netherlands Society for Oto-Rhino-Laryngology & Cervico-Facial Surgery. 2016 May 14.

184. van Beek SC, Conijn AP, Koelemay MJ, Balm R. Editor's Choice - Endovascular aneurysm repair versus open repair for patients with a ruptured abdominal aortic aneurysm: a systematic review and meta-analysis of short-term survival. European journal of vascular and endovascular surgery : the official journal of the European Society for Vascular Surgery. 2014 Jun;47(6):593-602.

185. van Bokhorst-de van der Schueren MA, Guaitoli PR, Jansma EP, de Vet HC. A systematic review of malnutrition screening tools for the nursing home setting. Journal of the American Medical Directors Association. 2014 Mar;15(3):171-84.

186. van Bokhorst-de van der Schueren MA, Guaitoli PR, Jansma EP, de Vet HC. Nutrition screening tools: does one size fit all? A systematic review of screening tools for the hospital setting. Clinical nutrition (Edinburgh, Scotland). 2014 Feb;33(1):39-58.

187. van Bunderen CC, van Varsseveld NC, Erfurth EM, Ket JC, Drent ML. Efficacy and safety of growth hormone treatment in adults with growth hormone deficiency: a systematic review of studies on morbidity. Clinical endocrinology. 2014 Jul;81(1):1-14.

188. van Dalen EC, Mank A, Leclercq E, Mulder RL, Davies M, Kersten MJ, et al. Low bacterial diet versus control diet to prevent infection in cancer patients treated with chemotherapy causing episodes of neutropenia. The Cochrane database of systematic reviews. 2016;4:CD006247.

189. van Dalen EC, van der Pal HJ, Kremer LC. Different dosage schedules for reducing cardiotoxicity in people with cancer receiving anthracycline chemotherapy. The Cochrane database of systematic reviews. 2016;3:CD005008.

190. van Dalen-Kok AH, Pieper MJ, de Waal MW, Lukas A, Husebo BS, Achterberg WP. Association between pain, neuropsychiatric symptoms, and physical function in dementia: a systematic review and meta-analysis. BMC geriatrics. 2015;15:49.

191. van de Glind EM, Rhodius-Meester HF, Reitsma JB, Hooft L, van Munster BC. Reviews of individual patient data (IPD) are useful for geriatrics: an overview of available IPD reviews. Journal of the American Geriatrics Society. 2014 Jun;62(6):1133-8.

192. van de Vorst IE, Koek HL, de Vries R, Bots ML, Reitsma JB, Vaartjes I. Effect of Vascular Risk Factors and Diseases on Mortality in Individuals with Dementia: A Systematic Review and Meta-Analysis. Journal of the American Geriatrics Society. 2016 Jan;64(1):37-46.

193. van den Berg JW, de Nier LM, Kaper NM, Schilder AG, Venekamp RP, Grolman W, et al. Limited evidence: higher efficacy of nasal saline irrigation over nasal saline spray in chronic rhinosinusitis--an update and reanalysis of the evidence base. Otolaryngology--head and neck surgery : official journal of American Academy of Otolaryngology-Head and Neck Surgery. 2014 Jan;150(1):16-21.

194. van den Broek MF, Gudden C, Kluijfhout WP, Stam-Slob MC, Aarts MC, Kaper NM, et al. No evidence for distinguishing bacterial from viral acute rhinosinusitis using symptom duration and purulent rhinorrhea: a systematic review of the evidence base. Otolaryngology--head and neck surgery : official journal of American Academy of Otolaryngology-Head and Neck Surgery. 2014 Apr;150(4):533-7.

195. van den Dungen P, van Kuijk L, van Marwijk H, van der Wouden J, Moll van Charante E, van der Horst H, et al. Preferences regarding disclosure of a diagnosis of dementia: a systematic review. International psychogeriatrics / IPA. 2014 Oct;26(10):1603-18.

196. van den Haak L, Alleblas C, Nieboer TE, Rhemrev JP, Jansen FW. Efficacy and safety of uterine manipulators in laparoscopic surgery: a review. Archives of gynecology and obstetrics. 2015 Nov;292(5):1003-11.

197. van der Gijp A, Ravesloot CJ, Jarodzka H, van der Schaaf MF, van der Schaaf IC, van Schaik JP, et al. How visual search relates to visual diagnostic performance: a narrative systematic review of eye-tracking research in radiology. Advances in health sciences education : theory and practice. 2016 Jul 19.

198. van der Have M, van der Aalst KS, Kaptein AA, Leenders M, Siersema PD, Oldenburg B, et al. Determinants of health-related quality of life in Crohn's disease: a systematic review and meta-analysis. Journal of Crohn's & colitis. 2014 Feb;8(2):93-106.

199. van der Heijden EH, Casal RF, Trisolini R, Steinfort DP, Hwangbo B, Nakajima T, et al. Guideline for the acquisition and preparation of conventional and endobronchial ultrasound-guided transbronchial needle aspiration specimens for the diagnosis and molecular testing of patients with known or suspected lung cancer. Respiration; international review of thoracic diseases. 2014;88(6):500-17.

200. van der Horst N, van de Hoef S, Reurink G, Huisstede B, Backx F. Return to Play After Hamstring Injuries: A Qualitative Systematic Review of Definitions and Criteria. Sports medicine (Auckland, NZ). 2016 Jun;46(6):899-912.

201. van der Linden MM, van Rappard DC, Daams JG, Sprangers MA, Spuls PI, de Korte J. Health-related quality of life in patients with cutaneous rosacea: a systematic review. Acta dermato-venereologica. 2015 Apr;95(4):395-400.

202. van der Meij E, Anema JR, Otten RH, Huirne JA, Schaafsma FG. The Effect of Perioperative E-Health Interventions on the Postoperative Course: A Systematic Review of Randomised and Non-Randomised Controlled Trials. PLoS One. 2016;11(7):e0158612.

203. van der Pols-Vijlbrief R, Wijnhoven HA, Schaap LA, Terwee CB, Visser M. Determinants of protein-energy malnutrition in community-dwelling older adults: a systematic review of observational studies. Ageing research reviews. 2014 Nov;18:112-31.

204. van der Steen JT, van Soest-Poortvliet MC, Hallie-Heierman M, Onwuteaka-Philipsen BD, Deliens L, de Boer ME, et al. Factors associated with initiation of advance care planning in dementia: a systematic review. Journal of Alzheimer's disease : JAD. 2014;40(3):743-57.

205. van der Voort P, Pijls BG, Nieuwenhuijse MJ, Jasper J, Fiocco M, Plevier JW, et al. Early subsidence of shape-closed hip arthroplasty stems is associated with late revision. A systematic review and meta-analysis of 24 RSA studies and 56 survival studies. Acta orthopaedica. 2015;86(5):575-85.

206. van Dongen RM, Zielman R, Noga M, Dekkers OM, Hankemeier T, van den Maagdenberg AM, et al. Migraine biomarkers in cerebrospinal fluid: A systematic review and meta-analysis. Cephalalgia : an international journal of headache. 2016 Feb 17.

207. van Drongelen J, de Vries R, Lotgering FK, Smits P, Spaanderman ME. Functional vascular changes of the kidney during pregnancy in animals: a systematic review and meta-analysis. PLoS One. 2014;9(11):e112084.

208. van Duijn JG, Isfordink LM, Nij Bijvank JA, Stapper CW, van Vuren AJ, Wegner I, et al. Rapid Systematic Review of the Epley Maneuver for Treating Posterior Canal Benign Paroxysmal Positional Vertigo. Otolaryngology--head and neck surgery : official journal of American Academy of Otolaryngology-Head and Neck Surgery. 2014 Mar 31;150(6):925-32.

209. van Erp WS, Lavrijsen JC, van de Laar FA, Vos PE, Laureys S, Koopmans RT. The vegetative state/unresponsive wakefulness syndrome: a systematic review of prevalence studies. European journal of neurology. 2014 Nov;21(11):1361-8.

210. van Galen KP, Engelen ET, Mauser-Bunschoten EP, van Es RJ, Schutgens RE. Antifibrinolytic therapy for preventing oral bleeding in patients with haemophilia or Von Willebrand disease undergoing minor oral surgery or dental extractions. The Cochrane database of systematic reviews. 2015(12):CD011385.

211. van Haalen FM, Broersen LH, Jorgensen JO, Pereira AM, Dekkers OM. Management of endocrine disease: Mortality remains increased in Cushing's disease despite biochemical remission: a systematic review and meta-analysis. European journal of endocrinology / European Federation of Endocrine Societies. 2015 Apr;172(4):R143-9.

212. van Herwaarden YJ, Verstegen MH, Dura P, Kievit W, Drenth JP, Dekker E, et al. Low prevalence of serrated polyposis syndrome in screening populations: a systematic review. Endoscopy. 2015 Nov;47(11):1043-9.

213. van Hulst AM, Kroon W, van der Linden ES, Nagtzaam L, Ottenhof SR, Wegner I, et al. Grade of dysplasia and malignant transformation in adults with premalignant laryngeal lesions. Head & neck. 2016 Apr;38 Suppl 1:E2284-90.

214. van Hulsteijn LT, Niemeijer ND, Dekkers OM, Corssmit EP. (131)I-MIBG therapy for malignant paraganglioma and phaeochromocytoma: systematic review and meta-analysis. Clinical endocrinology. 2014 Apr;80(4):487-501.

215. van Leeuwen MC, Stokmans SC, Bulstra AE, Meijer OW, Heymans MW, Ket JC, et al. Surgical Excision with Adjuvant Irradiation for Treatment of Keloid Scars: A Systematic Review. Plastic and reconstructive surgery Global open. 2015 Jul;3(7):e440.

216. van Litsenburg RR, Kunst A, Huisman J, Ket JC, Kaspers GJ, Gemke RJ. Health status utilities in pediatrics: a systematic review of acute lymphoblastic leukemia. Medical decision making : an international journal of the Society for Medical Decision Making. 2014 Jan;34(1):21-32.

217. van Loon MC, Merkus P, Smit CF, Smits C, Witte BI, Hensen EF. Stapedotomy in cochlear implant candidates with far advanced otosclerosis: a systematic review of the literature and meta-analysis. Otology & neurotology : official publication of the American Otological Society, American Neurotology Society [and] European Academy of Otology and Neurotology. 2014 Dec;35(10):1707-14.

218. van Meurs HS, van Lonkhuijzen LR, Limpens J, van der Velden J, Buist MR. Hormone therapy in ovarian granulosa cell tumors: a systematic review. Gynecologic oncology. 2014 Jul;134(1):196-205.

219. van Nies JA, Krabben A, Schoones JW, Huizinga TW, Kloppenburg M, van der Helm-van Mil AH. What is the evidence for the presence of a therapeutic window of opportunity in rheumatoid arthritis? A systematic literature review. Annals of the rheumatic diseases. 2014 May;73(5):861-70.

220. van Nieuwenhuizen AJ, Buffart LM, Brug J, Leemans CR, Verdonck-de Leeuw IM. The association between health related quality of life and survival in patients with head and neck cancer: a systematic review. Oral oncology. 2015 Jan;51(1):1-11.

221. van Oldenrijk J, Molleman J, Klaver M, Poolman RW, Haverkamp D. Revision rate after short-stem total hip arthroplasty: a systematic review of 49 studies. Acta orthopaedica. 2014 Jun;85(3):250-8.

222. van Oostendorp S, Elfrink A, Borstlap W, Schoonmade L, Sietses C, Meijerink J, et al. Intracorporeal versus extracorporeal anastomosis in right hemicolectomy: a systematic review and meta-analysis. Surgical endoscopy. 2016 Jun 10.

223. van Oostveen CJ, Ubbink DT, Huis in het Veld JG, Bakker PJ, Vermeulen H. Factors and models associated with the amount of hospital care services as demanded by hospitalized patients: a systematic review. PLoS One. 2014;9(5):e98102.

224. van Rijssen LB, Narwade P, van Huijgevoort NC, Tseng DS, van Santvoort HC, Molenaar IQ, et al. Prognostic value of lymph node metastases detected during surgical exploration for pancreatic or periampullary cancer: a systematic review and meta-analysis. HPB : the official journal of the International Hepato Pancreato Biliary Association. 2016 Jul;18(7):559-66.

225. van Tuyl LH, Lems WF, Boers M. Measurement of stiffness in patients with rheumatoid arthritis in low disease activity or remission: a systematic review. BMC musculoskeletal disorders. 2014;15:28.

226. van Velzen AS, Peters M, van der Bom JG, Fijnvandraat K. Effect of von Willebrand factor on inhibitor eradication in patients with severe haemophilia A: a systematic review. British journal of haematology. 2014 Aug;166(4):485-95.

227. van Vilsteren M, van Oostrom SH, de Vet HC, Franche RL, Boot CR, Anema JR. Workplace interventions to prevent work disability in workers on sick leave. The Cochrane database of systematic reviews. 2015(10):CD006955.

228. van Vlijmen EF, Wiewel-Verschueren S, Monster TB, Meijer K. Combined oral contraceptives, thrombophilia and the risk of venous thromboembolism: a systematic review and meta-analysis. Journal of thrombosis and haemostasis : JTH. 2016 Jul;14(7):1393-403.

229. van Wissen J, Bakker N, Doodeman HJ, Jansma EP, Bonjer HJ, Houdijk AP. Preoperative Methods to Reduce Liver Volume in Bariatric Surgery: a Systematic Review. Obes Surg. 2016 Feb;26(2):251-6.

230. van Zuuren EJ, Fedorowicz Z, Schoones J. Interventions for female pattern hair loss. The Cochrane database of systematic reviews. 2016(5):CD007628.

231. Veerbeek JM, van Wegen E, van Peppen R, van der Wees PJ, Hendriks E, Rietberg M, et al. What is the evidence for physical therapy poststroke? A systematic review and meta-analysis. PLoS One. 2014;9(2):e87987.

232. Vegting IL, Schrijver EJ, Otten RH, Nanayakkara PW. Internet programs targeting multiple lifestyle interventions in primary and secondary care are not superior to usual care alone in improving cardiovascular risk profile: a systematic review. European journal of internal medicine. 2014 Jan;25(1):73-81.

233. Vehmeijer JT, Brouwer TF, Limpens J, Knops RE, Bouma BJ, Mulder BJ, et al. Implantable cardioverter-defibrillators in adults with congenital heart disease: a systematic review and meta-analysis. European heart journal. 2016 May 7;37(18):1439-48.

234. Velzel J, de Hundt M, Mulder FM, Molkenboer JF, Van der Post JA, Mol BW, et al. Prediction models for successful external cephalic version: a systematic review. European journal of obstetrics, gynecology, and reproductive biology. 2015 Dec;195:160-7.

235. Verbeek-van Noord I, de Bruijne MC, Zwijnenberg NC, Jansma EP, van Dyck C, Wagner C. Does classroom-based Crew Resource Management training improve patient safety culture? A systematic review. SAGE open medicine. 2014;2:2050312114529561.

236. Verheggen RJ, Maessen MF, Green DJ, Hermus AR, Hopman MT, Thijssen DH. A systematic review and meta-analysis on the effects of exercise training versus hypocaloric diet: distinct effects on body weight and visceral adipose tissue. Obesity reviews : an official journal of the International Association for the Study of Obesity. 2016 Aug;17(8):664-90.

237. Verlinden CR, van de Vijfeijken SE, Jansma EP, Becking AG, Swennen GR. Complications of mandibular distraction osteogenesis for congenital deformities: a systematic review of the literature and proposal of a new classification for complications. International journal of oral and maxillofacial surgery. 2015 Jan;44(1):37-43.

238. Verloop WL, Beeftink MM, Santema BT, Bots ML, Blankestijn PJ, Cramer MJ, et al. A systematic review concerning the relation between the sympathetic nervous system and heart failure with preserved left ventricular ejection fraction. PLoS One. 2015;10(2):e0117332.

239. Verra WC, Boom LG, Jacobs WC, Schoones JW, Wymenga AB, Nelissen RG. Similar outcome after retention or sacrifice of the posterior cruciate ligament in total knee arthroplasty. Acta orthopaedica. 2015 Apr;86(2):195-201.

240. Vissenberg R, Manders VD, Mastenbroek S, Fliers E, Afink GB, Ris-Stalpers C, et al. Pathophysiological aspects of thyroid hormone disorders/thyroid peroxidase autoantibodies and reproduction. Human reproduction update. 2015 May-Jun;21(3):378-87.

241. Visser AW, Boyesen P, Haugen IK, Schoones JW, van der Heijde DM, Rosendaal FR, et al. Radiographic scoring methods in hand osteoarthritis--a systematic literature search and descriptive review. Osteoarthritis and cartilage / OARS, Osteoarthritis Research Society. 2014 Oct;22(10):1710-23.

242. Visser A, Geboers B, Gouma DJ, Goslings JC, Ubbink DT. Predictors of surgical complications: A systematic review. Surgery. 2015 Jul;158(1):58-65.

243. Vlek SL, Lier MC, Ankersmit MA, Ket JC, Dekker JJ, Mijatovic V, et al. Laparoscopic imaging techniques in endometriosis therapy - a systematic review. Journal of minimally invasive gynecology. 2016 Jul 5.

244. Vooijs M, Leensen MC, Hoving JL, Wind H, Frings-Dresen MH. Interventions to enhance work participation of workers with a chronic disease: a systematic review of reviews. Occupational and environmental medicine. 2015 Nov;72(11):820-6.

245. Vooijs M, Leensen MC, Hoving JL, Daams JG, Wind H, Frings-Dresen MH. Disease-generic factors of work participation of workers with a chronic disease: a systematic review. International archives of occupational and environmental health. 2015 Nov;88(8):1015-29.

246. Wang Y, Zekveld AA, Naylor G, Ohlenforst B, Jansma EP, Lorens A, et al. Parasympathetic Nervous System Dysfunction, as Identified by Pupil Light Reflex, and Its Possible Connection to Hearing Impairment. PLoS One. 2016;11(4):e0153566.

247. Weber MA, Limpens J, Roovers JP. Assessment of vaginal atrophy: a review. International urogynecology journal. 2015 Jan;26(1):15-28.

248. Wegner I, Niesten ME, van Werkhoven CH, Grolman W. Rapid Systematic Review of the Epley Maneuver versus Vestibular Rehabilitation for Benign Paroxysmal Positional Vertigo. Otolaryngology--head and neck surgery : official journal of American Academy of Otolaryngology-Head and Neck Surgery. 2014 May 20;151(2):201-7.

249. Wegner I, Kamalski DM, Tange RA, Vincent R, Stegeman I, van der Heijden GJ, et al. Laser versus conventional fenestration in stapedotomy for otosclerosis: a systematic review. The Laryngoscope. 2014 Jul;124(7):1687-93.

250. Wegner I, van den Berg JW, Smit AL, Grolman W. Systematic review of the use of bone cement in ossicular chain reconstruction and revision stapes surgery. The Laryngoscope. 2015 Jan;125(1):227-33.

251. Wegner I, van Waes AM, Bittermann AJ, Buitinck SH, Dekker CF, Kurk SA, et al. A Systematic Review of the Diagnostic Value of CT Imaging in Diagnosing Otosclerosis. Otology & neurotology : official publication of the American Otological Society, American Neurotology Society [and] European Academy of Otology and Neurotology. 2016 Jan;37(1):9-15.

252. Wuister AM, Goto NA, Oostveen EJ, de Jong WU, van der Valk ES, Kaper NM, et al. Nasal endoscopy is recommended for diagnosing adults with chronic rhinosinusitis. Otolaryngology--head and neck surgery : official journal of American Academy of Otolaryngology-Head and Neck Surgery. 2014 Mar;150(3):359-64.

253. Yauw ST, Wever KE, Hoesseini A, Ritskes-Hoitinga M, van Goor H. Systematic review of experimental studies on intestinal anastomosis. The British journal of surgery. 2015 Jun;102(7):726-34.

254. Zaal IJ, Devlin JW, Peelen LM, Slooter AJ. A systematic review of risk factors for delirium in the ICU. Critical care medicine. 2015 Jan;43(1):40-7.

255. Zaal-Schuller IH, de Vos MA, Ewals FV, van Goudoever JB, Willems DL. End-of-life decision-making for children with severe developmental disabilities: The parental perspective. Research in developmental disabilities. 2016 Feb-Mar;49-50:235-46.

256. Zandbelt LC, de Kanter FE, Ubbink DT. E-consulting in a medical specialist setting: Medicine of the future? Patient education and counseling. 2016 May;99(5):689-705.

257. Zijlstra IA, Verbaan D, Majoie CB, Vandertop P, van den Berg R. Coiling and clipping of middle cerebral artery aneurysms: a systematic review on clinical and imaging outcome. Journal of neurointerventional surgery. 2016 Jan;8(1):24-9.

258. Zwinkels M, Verschuren O, Janssen TW, Ketelaar M, Takken T. Exercise training programs to improve hand rim wheelchair propulsion capacity: a systematic review. Clinical rehabilitation. 2014 Sep;28(9):847-61.
